# Modelling the Role of the Hsp70/Hsp90 System in the Maintenance of Protein Homeostasis

**DOI:** 10.1371/journal.pone.0022038

**Published:** 2011-07-14

**Authors:** Carole J. Proctor, Ian A. J. Lorimer

**Affiliations:** 1 Centre for Integrated Systems Biology of Ageing and Nutrition, Institute for Ageing and Health, Newcastle University, Newcastle upon Tyne, United Kingdom; 2 Ottawa Hospital Research Institute, Ottawa, Canada; 3 Department of Biochemistry, Microbiology and Immunology, University of Ottawa, Ottawa, Canada; Michigan State University, United States of America

## Abstract

Neurodegeneration is an age-related disorder which is characterised by the accumulation of aggregated protein and neuronal cell death. There are many different neurodegenerative diseases which are classified according to the specific proteins involved and the regions of the brain which are affected. Despite individual differences, there are common mechanisms at the sub-cellular level leading to loss of protein homeostasis. The two central systems in protein homeostasis are the chaperone system, which promotes correct protein folding, and the cellular proteolytic system, which degrades misfolded or damaged proteins. Since these systems and their interactions are very complex, we use mathematical modelling to aid understanding of the processes involved. The model developed in this study focuses on the role of Hsp70 (IPR00103) and Hsp90 (IPR001404) chaperones in preventing both protein aggregation and cell death. Simulations were performed under three different conditions: no stress; transient stress due to an increase in reactive oxygen species; and high stress due to sustained increases in reactive oxygen species. The model predicts that protein homeostasis can be maintained during short periods of stress. However, under long periods of stress, the chaperone system becomes overwhelmed and the probability of cell death pathways being activated increases. Simulations were also run in which cell death mediated by the JNK (P45983) and p38 (Q16539) pathways was inhibited. The model predicts that inhibiting either or both of these pathways may delay cell death but does not stop the aggregation process and that eventually cells die due to aggregated protein inhibiting proteasomal function. This problem can be overcome if the sequestration of aggregated protein into inclusion bodies is enhanced. This model predicts responses to reactive oxygen species-mediated stress that are consistent with currently available experimental data. The model can be used to assess specific interventions to reduce cell death due to impaired protein homeostasis.

## Introduction

Cellular proteins are subject to oxidative damage and must either be repaired or removed to maintain protein homeostasis. Cells possess two main mechanisms to deal with damaged protein. The first mechanism is the chaperone system. Chaperones such as Hsp70 and Hsp90 bind to exposed hydrophobic regions of misfolded proteins and assist in the refolding process. Secondly, if refolding is unsuccessful, the misfolded protein is removed by the cellular proteolytic systems. The main system for removal of damaged and/or misfolded proteins is the proteasome, although autophagy also plays a role. If either the chaperone system or degradation pathways are perturbed, damaged protein may accumulate and start to form aggregates. Aggregated protein has been implicated in age-related neurodegenerative disorders although there is still controversy over whether it is a cause or consequence of the disease process [1]. Small aggregates bind to proteasomes but are unable to be degraded and so inhibit proteasomal function [2]. This will lead to a reduction in the clearance of misfolded protein and a vicious cycle might ensue.

Levels of reactive oxygen species (ROS) increase with age due to either an increase in dysfunctional mitochondria or a decline in the antioxidant system [3], [4] leading to increase damage to cellular components. In addition it has been observed that there is a progressive decline in the overall proteolytic capacity of the cell with age [5], [6] resulting in an accumulation of oxidized and cross-linked proteins [7], [8]. Chaperone function has also been shown to decline with age [9], [10]. There are several links between the chaperone and degradation pathways. For example, the molecular co-chaperone CHIP (C terminus of Hsc70-interacting protein, (Q9UNE7)) serves as a catalyst for the ubiquitination of several Hsp70 and Hsp90 client proteins that have been linked to neurodegenerative disorders [11]. Therefore, interventions which target part of one pathway will also impact the rest of the system.

A stochastic model of the chaperone Hsp90 was previously developed to examine the role of chaperones in the ageing process [12]. This model showed that under conditions of low or transient stress, chaperone capacity is sufficient to maintain protein homeostasis. Even under conditions of increasing stress with age, normal chaperone capacity is able to deal with the increasing overload due to the mechanism of upregulation of Hsps after stress. However, if the model includes a decline in chaperone function with age, then the system becomes overwhelmed and protein homeostasis is lost. This model only included Hsp90 and one client of Hsp90, namely Heat Shock Factor-1 (Hsf1, (Q00613)) but was constructed in the Systems Biology Mark-up Language (SBML) [13] which allows easy extension as required.

The role of apoptosis in ageing has received considerable interest but the exact relationship between ageing and apoptosis has yet to be established [14]. Apoptosis has a critical role in tissue homeostasis, and in mitotic tissue is important for preventing tumorigenesis. In post-mitotic tissue, ageing is associated with increased apoptosis to remove dysfunctional cells and since these cells are irreplaceable, this will have an impact on tissue function. For example, neuronal loss is a common feature of neurodegeneration and results in pathology such as memory loss, movement disorders and/or visual impairment, depending on the specific part of the brain which is affected. Therefore it is important to extend the model to include apoptotic pathways. Many chaperones are involved in apoptotic pathways such as Hsp27 and Hsp70 which are antiapoptotic. There are many pathways involved in apoptosis but our model will concentrate on three pathways that are particularly relevant to neurodegeneration. The first pathway involves the c-Jun N-terminal kinase (JNK). JNK (MAPK8) is activated by phosphorylation and in turn, JNK phosphorylates a number of proteins involved in various signalling pathways including apoptosis. It is maintained in an inactive state by the phosphatase Mkp1 (DUSP1, (P28562)) which requires Hsp70 for its activation. When levels of free Hsp70 are high, Mkp1 is activated and JNK activation is inhibited. Under conditions of stress, misfolded protein binds to Hsp70, diminishing pools of free Hsp70, so less Mkp1 is activated. Therefore, JNK remains phosphorylated and can induce one of the signalling pathways which lead to apoptosis. In this model we assume that the probability of cell death depends on the level of activated JNK, with the understanding that future models could incorporate more additional regulatory elements. Upregulation of Hsp70 by the stress response maintains pools of free Hsp70 to prevent activation of this apoptotic pathway. Mkp1 is prone to oxidation in its catalytic domain resulting in inactivation of its phosphatase activity [15]. This means that ROS elevation has opposing effects on Mkp1 activity. On the one hand, increased pools of Hsp70 leads to activation of Mkp1, but on the other hand oxidation of Mkp1 by ROS leads to its inactivation. These opposing effects of ROS are incorporated into the model.

The second apoptotic pathway considered involves the mitogen-activated protein kinase p38 (MAPK14). It has been shown that p38 is activated by an increase in levels of ROS [16], and that p38 is also involved in generating ROS, so providing a positive feedback loop [16], [17], [18]. Similarly to JNK, p38 is de-phosphorylated by Mkp1 to keep it in an inactive state. As for JNK, we model this apoptotic pathway by assuming that the probability of cell death depends on the level of activated p38. The third apoptotic pathway that we include involves the transcription factor p53 (P04637) which is normally present in low levels as it is rapidly turned over by the ubiquitin-proteasome pathway. However, if the proteasome is inhibited, levels of p53 will rise and either cell cycle arrest (relevant only for dividing cells) or apoptosis takes place. We do not include details of the p53 apoptotic pathway in this model as this pathway is very complex and would also have needed to include detail of p53 turnover. This extra complexity would have made stochastic simulations and model analysis impractical to do in terms of the computer simulation time required. Therefore, for simplicity we assume that the probability of cell death depends on the level of aggregated protein inhibiting the proteasome.

Akt (P31749) is a kinase which plays an important role in cellular signalling and it is involved in survival pathways. It is a client of Hsp90 and is destabilised by inhibitors of Hsp90 function [19]. It phosphorylates glycogen synthase kinase-3 beta (GSK3β, (P49841)) [20], one of the main kinases responsible for tau (P10636) hyperphosphorylation. A model of tau aggregation is also being developed with the intention of linking it to this model of chaperones and so we include Akt as a common link for these models. Hsp90 inhibitors, such as geldamycin, bind tightly to the Hsp90 ATP/ADP pocket and prevent ATP binding and the completion of client protein refolding. Instead proteins are degraded. For example, Basso *et al.* [19] found that Hsp90 inhibitors do not alter association of Akt with Hsp90 but result in ubiquitination of Akt and its subsequent degradation. They found that the half-life of Akt was shortened from 36 hours to 12 hours in cells exposed to the Hsp90 inhibitor 17-AAG.

Here we have extended the chaperone model of Proctor *et al.* [12] to include details of Hsp70 and its role in apoptosis, as well as its chaperoning activity. We also include detail of some of the client proteins of Hsp70 and Hsp90. We hypothesize that: (1) under normal conditions, basal levels of chaperones are able to maintain protein homeostasis; (2) under conditions of low or moderate stress, there is a transient increase in protein misfolding but chaperones are upregulated and protein homeostasis is restored; and (3) under conditions of prolonged high stress, the chaperone system is overwhelmed and apoptosis takes place. We now give more details of each of these scenarios.

### (1) Normal conditions -unstressed cells

**Figure 1:**
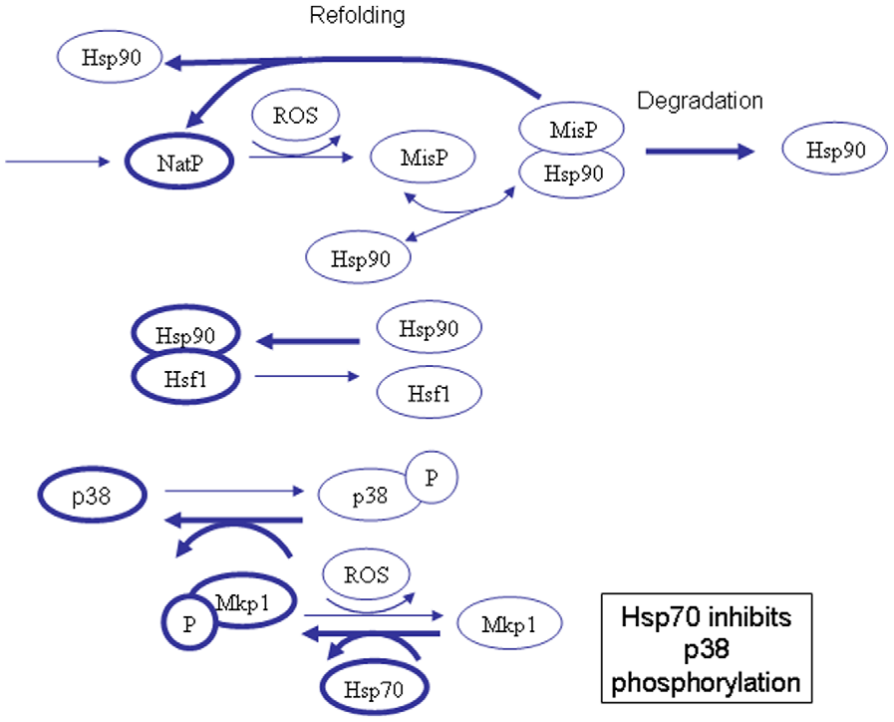
Network structure of model for unstressed cells. Under normal conditions, free pools of Hsp70 and Hsp90 are high and pools of misfolded protein are low. Hsp90 is bound to Hsf1 to prevent transcription of heat shock genes. Hsp70 activates Mkp1 which leads to dephosphorylation of JNK and p38, so that apoptotic pathways are not activated. Thickness of lines indicates most likely reactions and states.

**Figure 2:**
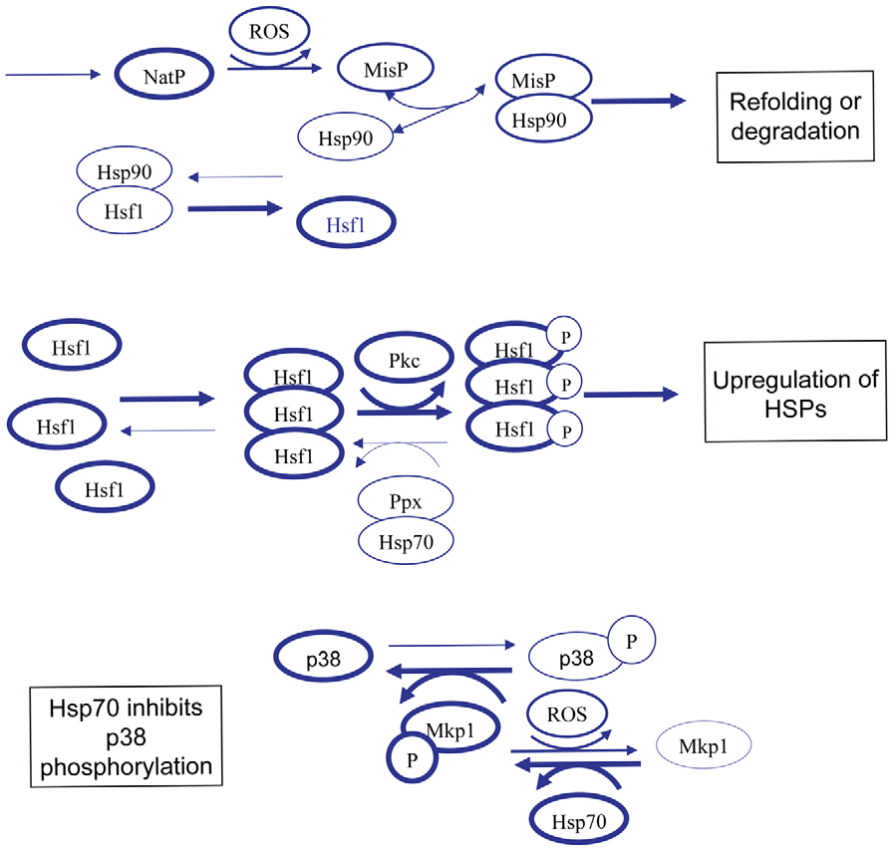
Network structure of model for low or moderate levels of stress. Under conditions of low or moderate stress, pools of misfolded protein increase and bind to Hsp70 and Hsp90 in competition with other substrates. This leads to increased pools of Hsf1 which can now form trimers. The trimers are then activated resulting in transcription of Hsps. Since pools of Hsp70 are increased, there is still sufficient Hsp70 to prevent apoptosis.

**Figure 3:**
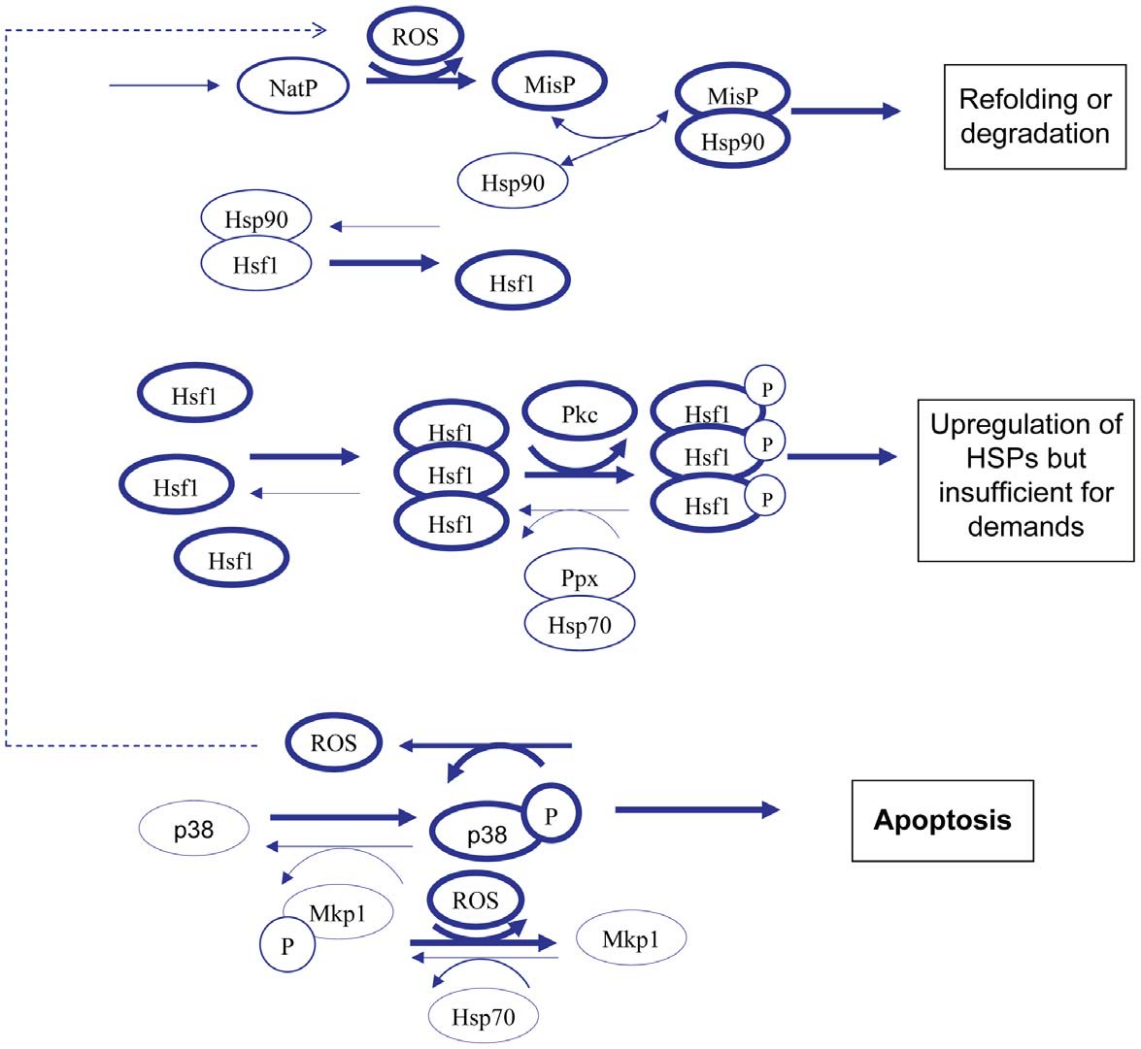
Network structure of model for high levels of stress. Under conditions of high or prolonged stress, high levels of misfolded protein may overwhelm the chaperone system. Although upregulation of Hsps takes place, pools of Hsp70 may be insufficient to activate Mkp1 and so there is a high probability that apoptosis will take place.

Under normal cellular conditions (Figure 1) there are very low levels of damaged/misfolded protein which are either refolded via the chaperone system or eliminated from the cell via the ubiquitin-proteasome system. The majority of Hsf1 is bound to Hsp90 which prevents Hsf1 from becoming transcriptionally active. There is basal transcription of heat shock proteins to maintain pools at a steady state level but there is no upregulation of Hsps. JNK and p38 are maintained in their unphosphorylated state due to activity of the phosphatase Mkp1, which requires Hsp70 for its activity. Since there are sufficient pools of free Hsp70, apoptosis is inhibited. Figure 1 omits detail of JNK for clarity. (Note that Figures 1, 2, 3 are biological diagrams that summarise the key components of the model. A diagram of the full model network is shown in Figure S1).

### (2) Low – moderate levels of stress

When cells are stressed (Figure 2), damaged/misfolded proteins accumulate and bind to Hsp70 and Hsp90. Once bound to chaperones, they may be refolded or sent for degradation. Binding of misfolded proteins to chaperones also prevents their aggregation. Due to competition from misfolded proteins, Hsf1 is released from Hsp90 and its concentration is now sufficient for it to form trimers. Hsf1 trimers are phosphorylated and become transcriptionally active so Hsps are upregulated. Hsp70 also binds to a phosphatase (currently unknown and so is referred to as PPX) which will dephosphorylate the trimers. This occurs when the concentration of free Hsp70 is sufficiently high and provides a negative feedback loop to prevent further upregulation of Hsps when they are present in sufficient number. This also provides a mechanism to switch off transcription of Hsps once the stress is over. There is sufficient Hsp70, despite some of the pool binding to misfolded protein, to continue to keep Mkp1 active and so prevent apoptosis.

### (3) High levels of stress

If cells are subject to high levels of stress over long time periods (Figure 3), there will be a large increase in misfolded proteins which bind to the pool of free Hsp70 and Hsp90. Upregulation of Hsps will occur but eventually the chaperone system is overwhelmed and free pools are severely depleted. The idea of an overload of the chaperone system has been previously proposed and so we will use our model to test this hypothesis [21]. There is also experimental evidence that chaperone systems are overwhelmed during periods of high stress. For example, Sangster *et al*. have shown that elevated temperature can phenocopy the effects of Hsp90 inhibition on genetic trait selection in *Drosophila*. This is a consequence of heat-induced widespread misfolding of proteins overwhelming the Hsp90 chaperone system and thereby preventing it from assisting in a subset of proteins that require Hsp90 to fold correctly under non-stressed conditions [22]. Cowen and Lindquist have made similar observations in yeast cells [23]. An overload of the chaperone system leads to depletion of free pools of Hsp70 which allows phosphorylation of JNK and p38 and subsequently leads to apoptosis. There may also be an increase in aggregated protein if there is insufficient Hsp70 and Hsp90 to bind to the misfolded proteins. In addition, activation of p38 leads to increased ROS and so even more protein misfolding occurs. It has been shown that damaged protein accumulates with age in post-mitotic cells, and in addition, there is also an age-related increase in dysfunctional mitochondria leading to increased levels of ROS. All these factors may explain why the risk of neurodegenerative disorders increases with age.

## Results

The purpose of building the model is to test the following hypotheses: (1) protein homeostasis is maintained during periods of low or moderate levels of stress; (2) protein homeostasis is disturbed after prolonged high levels of stress leading to cell death due to an overload of the chaperone system. Therefore we ran the model under three different scenarios: normal conditions (no stress); a transient increase in ROS (moderate stress); and with increasing stress with time (high stress).

### Normal conditions (no stress)

**Figure 4:**
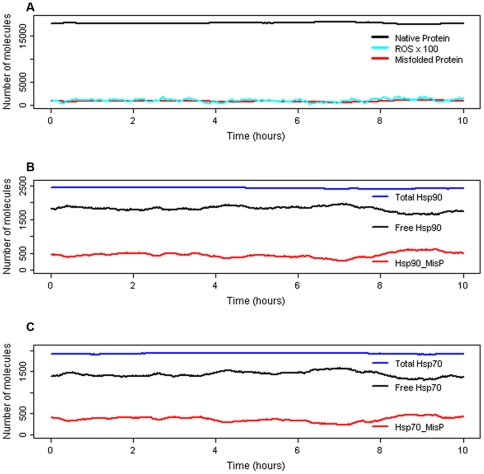
Simulation results for normal conditions. One typical simulation result showing levels of some of the model species. A Native protein, total misfolded protein (includes misfolded bound by Hsps), and reactive oxygen species (ROS). ROS are scaled x100 to allow easier visualisation. B Total Hsp90 (free pools plus all complexes), Free Hsp90 (unbound Hsp90) and Hsp90_MisP (Hsp90 bound to misfolded protein). C Total Hsp70 (free pools plus all complexes), Free Hsp70 (unbound Hsp70) and Hsp70_MisP (Hsp70 bound to misfolded protein).

We set the model parameters so that under normal conditions levels of all proteins remain fairly constant, there is no upregulation of Hsps and no activation of JNK or p38. A typical simulation result is shown in Figure 4. As we have used stochastic simulation, we carried out multiple runs and plotted the mean values for 100 simulations (Figure S2). The model for unstressed conditions was also run in a deterministic simulator and produces very similar results to the stochastic model (Figure S3).

### Transient increase in ROS (moderate stress)

**Figure 5:**
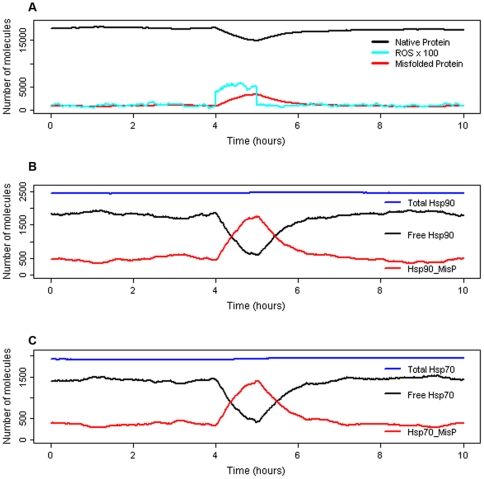
Simulation results for moderate/transient stress. ROS levels were increased by a factor of 4 at time t = 4 hours for a period of 2 hours. One typical simulation result is shown. A Native protein, total misfolded protein (includes misfolded bound by Hsps), and reactive oxygen species (ROS). ROS are scaled x100 to allow easier visualisation. B Total Hsp90 (free pools plus all complexes), Free Hsp90 (unbound Hsp90) and Hsp90_MisP (Hsp90 bound to misfolded protein). C Total Hsp70 (free pools plus all complexes), Free Hsp70 (unbound Hsp70) and Hsp70_MisP (Hsp70 bound to misfolded protein).

We ran stochastic simulations in which we increased ROS levels four-fold for a period of 1 hour (Figure 5). Deterministic simulation produced similar results to the stochastic model (Figure S4). After ROS levels increase (at time = 4 hours), there is an increase in misfolded protein which binds to Hsp70 and Hsp90. The model predicts that initially there are sufficient pools of unbound Hsps and so clients including Hsf1 remain bound to Hsp70 and Hsp90. As pools of unbound Hsps become depleted, misfolded protein competes with the client proteins but very little Hsf1 is released from its complex since the binding affinity of Hsf1 to Hsp90 is much greater than the binding affinity of other clients. So the model predicts only a very small increase in total Hsp levels. The misfolded protein is either refolded or degraded and replaced with newly synthesised protein so that by 2 hours after ROS levels have returned to normal, protein homeostasis is restored. We also looked at the effect in varying the amount and duration of stress by modifying the events for increasing and decreasing stress so that ROS was increased by a factor of 2, 4 or 8 for a period of 1, 2, 3 or 4 hours. The time to recovery under each condition was calculated and was assumed to be the time taken for the total level of misfolded protein after the stress event to return to within one standard deviation of the mean level of misfolded protein before the stress event. The model predicts that increasing the duration of stress leads to a shorter time of recovery for all stress levels (Table 1). This was due to greater upregulation of Hsps which persisted even after the stress was over. Increasing the amount of stress from two-fold to four-fold increased the amount of misfolded protein but also increased the amount of heat shock proteins so that the time for recovery was also shortened. However, an 8-fold increase in the amount of ROS led to longer recovery times compared to a 4-fold increase when the duration of stress was 1, 2 or 3 hours. This was due to a much larger increase in misfolded proteins which outnumbered the pool of heat shock proteins.

**Table 1:** Simulation results of varying the amount and duration of a transient increase in ROS levels.

Duration of stress (h)	Fold increase	Time for recovery (h)	Maximum level of misfolded protein	Maximum level of total Hsps
1	2	3.24	1554.3	4377.2
2	2	2.76	1682.9	4383.6
3	2	2.46	1699.3	4394.4
4	2	2.27	1699.3	4404.5
1	4	3.06	2771.6	4398.9
2	4	2.69	3082.5	4435.8
3	4	2.43	3101.1	4470.8
4	4	2.24	3101.1	4503.6
1	8	3.12	5060.5	4467.1
2	8	3.02	6025.4	4713.0
3	8	2.74	6104.0	4939.1
4	8	2.06	6104.0	5030.8

### Increasing ROS with time (high stress)

**Figure 6:**
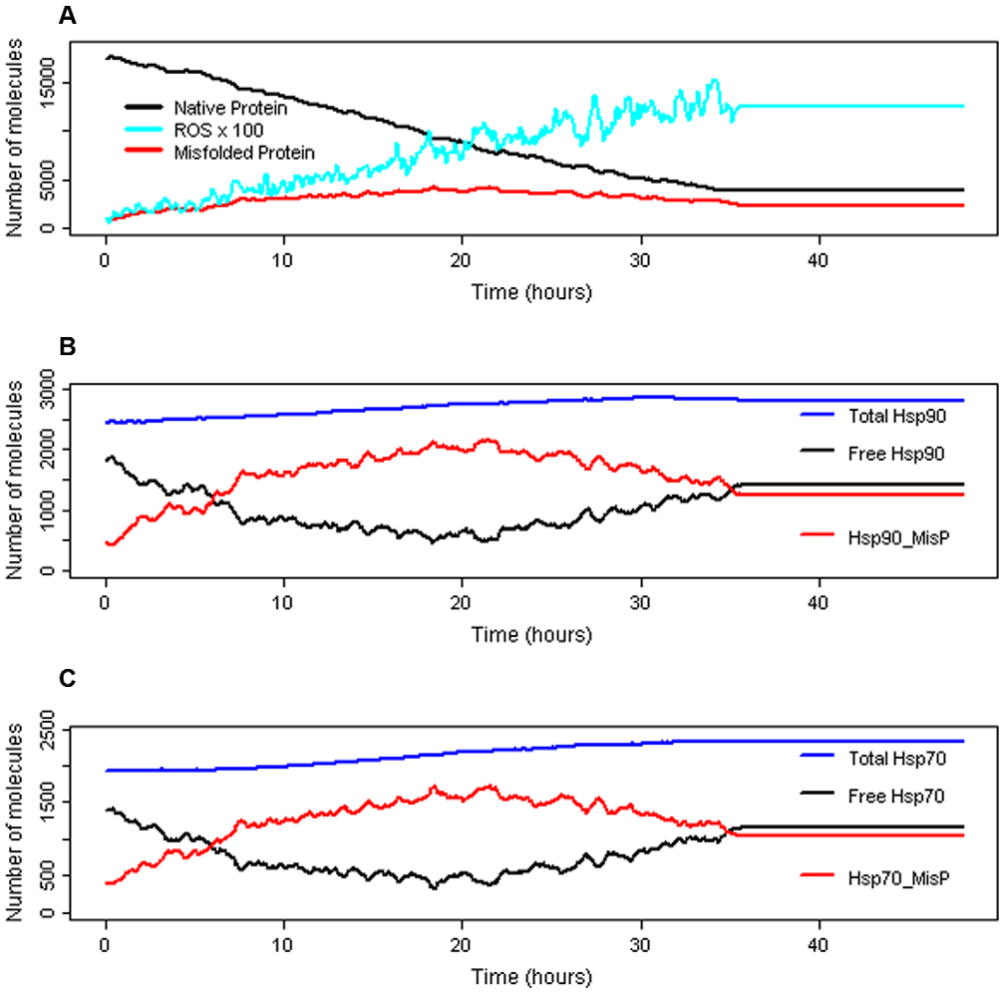
Simulation results for a continuous increase in ROS levels over time. The rate of ROS production was set to be an increasing function of time. One typical simulation showing a cell which underwent apoptosis at time t = 32.3 h. Note that the curves flatten after cell death as all reactions rates are set to zero. A Native protein, total misfolded protein (includes misfolded bound by Hsps), and reactive oxygen species (ROS). ROS are scaled x100 to allow easier visualisation. B Total Hsp90 (free pools plus all complexes), Free Hsp90 (unbound Hsp90) and Hsp90_MisP (Hsp90 bound to misfolded protein). C Total Hsp70 (free pools plus all complexes), Free Hsp70 (unbound Hsp70) and Hsp70_MisP (Hsp70 bound to misfolded protein).

We replaced a constant rate of ROS production with a rate that increases linearly with time (Figure S5). This was achieved by including the time variable in the rate law (see Methods). As the results for this situation are very variable it was necessary to do 100 simulations. We ran the simulation for 48 hours (simulated time) and the model predicted that 74% cells died during this period. The cause of death was recorded and in this set of simulations, 32% of cell deaths are due to the p38 pathway and 42% due to the JNK pathway. Since we assume that the reaction rates for both pathways are equal, we would expect the number of deaths by each pathway to be equal to 37. If we assume that the number of deaths in 100 simulations follows a Poisson distribution with mean 37, then the standard deviation is 6.1. So obtaining 32 and 42 cell deaths via the p38 and JNK pathways respectively is due to stochastic effects. Cell death takes place due to insufficient free Hsp70 for the activation of Mkp1 which is required to prevent phosphorylation of both JNK and p38. There are no cell deaths due to proteasome inhibition, as even in cells which survive, 48 hours is insufficient for aggregates to start accumulating. Cell death occurs at variable times ranging from 0.4 to 47.4 hours with the median time to death equal to 34.4 hours which corresponds to the time at which ROS levels reached about 10 times the basal level (Figure 6). The model predicts that as ROS levels increase, there is also an increase in misfolded protein and levels of total protein decrease due to an increase in degradation of misfolded protein. The pools of free Hsp70 and Hsp90 decline to low levels but then start to increase again due to upregulation of Hsps. However, there are insufficient chaperones to deal with the overwhelming burden of misfolded protein or to inhibit cell death pathways.

### Deterministic model – ROS increasing with time

Deterministic simulation for the model with increasing ROS with time was carried out using CellDesigner and COPASI. For this set of conditions the deterministic and stochastic models give incomparable results. The deterministic model predicts that the time to cell death is 38.8 h (Figure S6) and gives no information on the variability in the time to death. It should be noted that the way the deterministic simulator models cell death is quite different to the stochastic method. In the deterministic simulation, the species variable CellDeath represents the sum of p38Death, JNKDeath and PIDeath and gradually increases with time until it reaches a value of 1.0 when the simulation stops. In the stochastic simulation, CellDeath remains at zero until a cell death pathway is activated by p38, JNK or proteasome inhibition and then changes to 1.0 and the simulation stops. Despite these differences, the deterministic version is useful for looking at the effect of each parameter on the timing of cell death (see below).

### Inhibition of JNK and p38 cell death pathways

**Figure 7:**
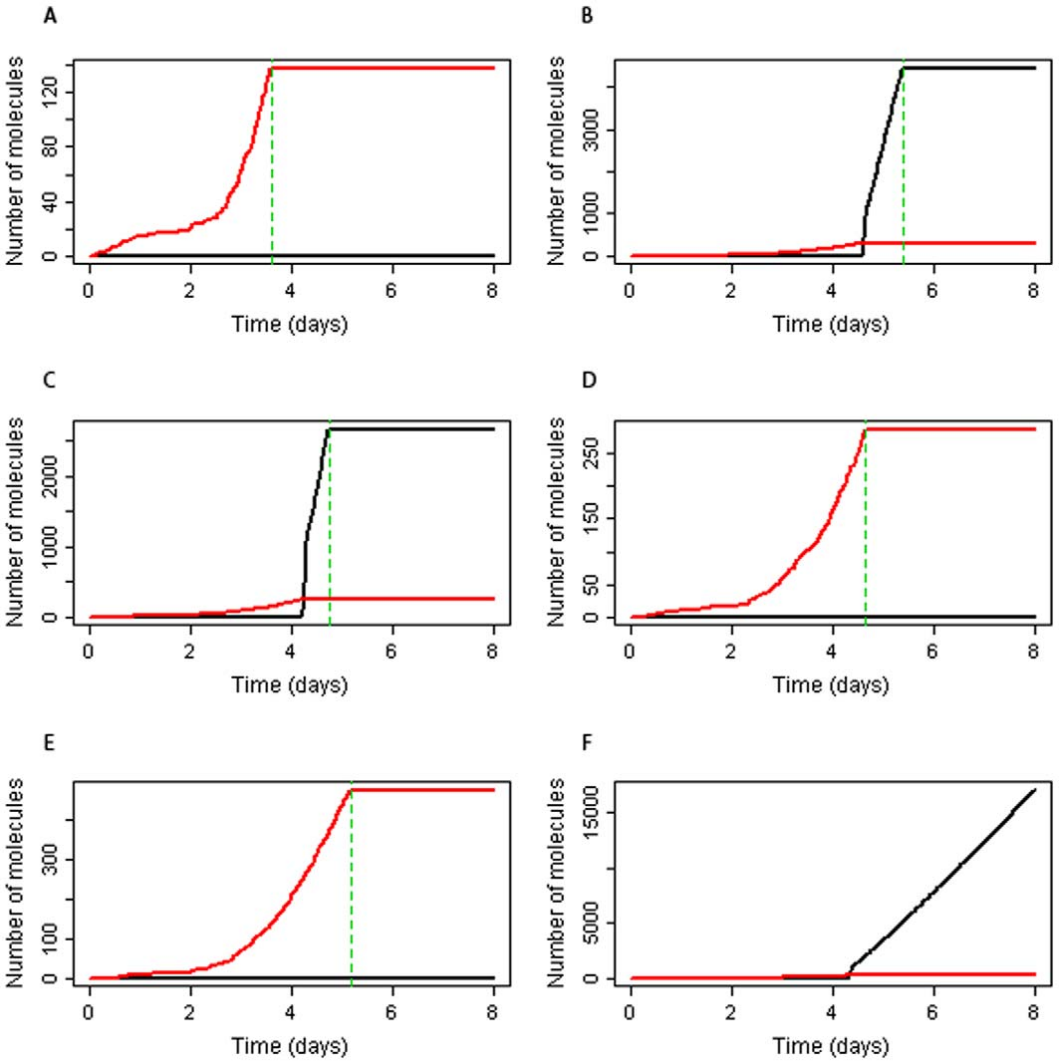
Simulation results for a continuous increase in ROS levels over time when p38 and JNK death pathways are inhibited. A–F Results from six different simulation runs. Aggregated protein sequestered into inclusion bodies (SeqAggP) and aggregated protein bound to proteasomes (AggP_Proteasome). Vertical dashed green line indicates time of cell death due to proteasome inhibition.

Simulations in which ROS levels increase with time were carried out with inhibition of cell death pathways via JNK and p38 in order to examine if protein aggregation occurs over longer time periods. The model predicts that aggregates start to form and bind to the proteasome at about 24 hours but it takes four or five days before levels at the proteasome are high enough to cause cell death (Figure 7). Cell death by 8 days occurs in about 80% of simulations, with the earliest time of death occurring at 55.7 hours and median time to death equal to about 5.7 days. Interestingly, in some simulation runs, aggregates are sequestered into inclusion bodies and if this starts to happen before aggregates bind to the proteasome, then all further aggregates get sequestered and cell death via proteasome inhibition is prevented (Figure 7F). If the parameter for upregulation of Hsps is increased, then the model predicts that aggregates take much longer to form as more misfolded proteins are able to bind to Hsp70 and Hsp90 which prevents their aggregation. Alternatively, if the parameter for sequestering of aggregates is increased by a factor of two or more, then proteasome inhibition is prevented. Therefore the model predicts that inhibition of cell death pathways via p38 or JNK may not be beneficial unless levels of misfolded and aggregated protein can also be reduced. Since *k_upregHsp_* and *k_seqagg_* have an effect on protein aggregation, we also checked whether changing these parameters had an effect on the time to cell death. In the model which includes JNK and p38 death pathways, neither parameter has an effect on the time to cell death. However, when both JNK and p38 death pathways are inhibited, varying *k_seqagg_*, from half to double its initial value, did affect the predicted time to cell death in the deterministic model (Figure S7). Varying *k_upregHsp_* over the same range had little effect on time to cell death, and it was necessary to decrease (and increase) its value over two orders of magnitude to see an effect (Figure S8).

### Effect of relative change in the JNK and p38 rate constants

We assumed that cell death pathways via p38 and JNK have equal probability of being activated and which pathway was activated was determined by stochastic effects. This may not be the case in reality and so we investigated the effect of relative changes in the JNK and p38 rate constants. We lowered *k_p38death_* by 50% and increased *k_Jnkdeath_* by 50% so that the overall death rate remained the same and carried out 100 simulations. The model predicts that 58 simulated cells die due to the JNK pathway, 22 simulated cells die due to the p38 pathway and 20 simulated cells remain alive by 48 hours. The median time to cell death is 27.3 hours (range 0.4–47.7 hours). The cells which are still alive are included in the calculation for the median since we assume that if we carried out simulations for longer time periods all cells would die and the time of later deaths does not affect the median value (provided more than 50% of cells have died) unlike the effect on the mean. The median time to cell death when the JNK and p38 constant rates are equal is 34.4 hours, so changing the relative rates results in earlier cell death. In addition, 100 simulations were carried out in which *k_p38death_* was increased by 50% and *k_Jnkdeat_*_h_ decreased by 50%. In this scenario, the model predicts 19 deaths due to JNK pathway, 64 deaths due to p38 pathway and 17 cells still surviving by 48 h with median time to cell death equal to 31.9 hours (range 0.4–47.7 hours). So again the model predicts earlier cell death if one pathway has a greater probability of being activated than when both death pathways have equal probabilities.

### Hsp90 inhibition

The use of Hsp90 inhibitors have been proposed as a potential beneficial therapy in neurodegenerative diseases (recently reviewed in [24]). Hsp90 inhibitors release Hsf1 from Hsp90 resulting in upregulation of heat shock proteins, and in addition they promote the degradation of Hsp90 clients. This works by blocking the ATPase activity of Hsp90 so that instead of clients being refolded they are targeted for degradation. As already mentioned, the half-life of Akt is reduced from about 36 hours to 12 hours after the addition of an Hsp90 inhibitor [19]. To model the effects of Hsp90 inhibition we set the parameters for Hsp90/Hsf1 binding and for the release of Hsp90 from its clients (Hsp90client and Akt) to zero after one hour of simulation time using an event structure (see Table 2). In order to check that Hsp90 affects the half-life of Akt we set the synthesis rate for Akt to zero and ran the model with and without Hsp90 inhibition. The model predicts that the half-life of Akt is reduced from 36.2 hours to 13.7 hours when Hsp90 is inhibited, in agreement with the experimental data. When we restore Akt synthesis, the model predicts a decline in Akt pools if Hsp90 is inhibited. There is also an increase in Hsp70 and Hsp90 pools due to the upregulation of heat shock proteins after Hsp90 inhibition. The effects of Hsp90 inhibition under conditions of high stress (ROS increases linearly with time) was also examined. In this case, the model predicts that there is no significant effect on the percentage of cell deaths or the timing of cell death. However, the current model does not specifically include Hsp90 clients such as GSK3β or mutant tau which like Akt are regulated by Hsp90 via CHIP. We would expect that inhibition of Hsp90 would lead to a decline in GSK3β and mutant tau levels and so we would predict a reduction in cell death. On the other hand Akt inhibits GSK3β activity and so GSK3β may become more active even if actual protein levels are reduced. Therefore it will be of interest to add further detail to the model to test these predictions.

**Table 2:** Model Events.

Name	Trigger	Assignments
DeathOfCell	CellDeath > = 1.0	*k_alive_* = 0
incROS	t>14400.0	ROS = 40; *k_genROS_* = 0.04
decROS	t>18000.0	ROS = 10; *k_genROS_* = 0.01
Hsp90Inhibition	t>3600.0	*k_binHsf1Hsp90_* = 0; *k_relHsp90client_* = 0; *k_relAktHsp90_* = 0

### Model analysis

#### 1. Parameter scan for model under normal conditions

Many of the parameters in the model are estimated based on knowledge of protein half-lives or on knowledge of the rate of different types of reactions in cells (e.g. phosphorylation occurs over a time-scale of minutes). Since it is not possible to obtain exact measurements for the parameters in this model we carried out a parameter scan to see which parameters affected the steady state of the system under normal conditions using a deterministic version of the model in COPASI [25]. Each parameter was scanned over ten values ranging from half to double its initial value. The results of the parameter scans indicate that altering values of the majority of parameters over a two-fold range have little effect on the steady state of the system under normal conditions. The parameters which did affect the system are shown in Table 3 and are those involved in protein turnover, misfolding and folding, basal turnover of Hsp70 and Hsp90, and turnover of ROS. The parameters which have the greatest effect on the levels of misfolded protein are *k_misfold_*, *k_refold_*, *k_genROS_* and *k_remROS_*. The model suggests that the best way to reduce levels of misfolded protein is to lower levels of ROS. The kinetics of p38 phosphorylation/de-phosphorylation also have a small effect on pools of NatP, MisP and free pools of Hsps due to its effect on ROS levels (Table 3). The parameters which had more than a 10% effect on protein pools were also examined using the stochastic model (Table 4). As it is necessary to do repeat simulations, we just examined the effect of doubling the value of each parameter and calculated the mean values from 20 repeat simulations. The results are very similar to the deterministic model (compare Tables 3 and 4).

**Table 3:** Results of parameter scan for deterministic model under normal conditions.

Parameter	NatP	Total MisP	Free pool of Hsp70	Free pool of Hsp90
*k_synNatP_*	**↑** 0.079	**↑** 0.075	↓ 0.018	↓ 0.018
*k_misfold_*	↓ **0.110**	↑ **0.798**	↓ **0.172**	↓**0.173**
*k_binMisPProt_*	↓ 0.058	↓**0.146**	↑ 0.037	↑ 0.036
*k_refold_*	↑ 0.060	↓ **0.432**	**↑** 0.096	**↑** 0.096
*k_basalsynHsp70_*	↔ 0.000	↓ 0.002	**↑ 0.160**	↑ 0.014
*k_basalsynHsp90_*	↔ 0.000	↓ 0.002	↑ 0.014	**↑ 0.131**
*k_binHsp70Prot_*	↔ 0.000	↑ 0.002	↓ **0.152**	↓ 0.015
*k_binHsp90Prot_*	↔ 0.000	↑ 0.002	↓ 0.016	↓ **0.131**
*k_genROS_*	↓ **0.108**	**↑ 0.799**	↓ **0.176**	**↓ 0.177**
*k_remROS_*	**↑** 0.064	↓ **0.481**	↑ **0.108**	**↑ 0.109**
*k_phosp38_*	↓ 0.005	↑ 0.033	↓ 0.007	↓ 0.007
*k_dephosp38Mkp1_*	**↑** 0.002	↓ 0.017	↑ 0.004	↑ 0.004

Each parameter was scanned over 10 values ranging from half to double its initial value over a period of 10 hours. The results show the effect of increasing the parameter from its initial value on the pools of NatP, MisP (including MisP bound to Hsps and the proteasome), free pools of Hsp70 and free pools of Hsp90. The arrows show the direction of change, the numbers indicate the proportional change in the species value when the parameter value is doubled (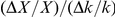), where the denominator is equal to one, if the parameter value *k* is double). Effects which are greater than 10% are indicated in boldface.

**Table 4:** Results of parameter scan for stochastic model under normal conditions.

Parameter	NatP	Total MisP	Free pool of Hsp70	Free pool of Hsp90
*k_misfold_*	↓ **0.114**	↑ **0.864**	↓ **0.172**	↓**0.173**
*k_binMisPProt_*	↓ 0.063	↓**0.173**	↑ 0.037	↑ 0.036
*k_refold_*	↑ 0.057	↓ **0.444**	**↑** 0.096	**↑** 0.096
*k_basalsynHsp70_*	↓ 0.006	↑0.027	**↑ 0.157**	↔ 0.000
*k_basalsynHsp90_*	↓ 0.006	↑0.027	↑ 0.014	**↑ 0.131**
*k_binHsp70Prot_*	↓ 0.006	↑ 0.032	↓ **0.164**	↓ 0.028
*k_binHsp90Prot_*	↓ 0.006	↑ 0.008	↓ 0.014	↓ **0.135**
*k_genROS_*	↓ **0.108**	**↑ 0.752**	↓ **0.164**	**↓ 0.169**
*k_remROS_*	**↑** 0.063	↓ **0.492**	↑ **0.107**	**↑ 0.107**

Each parameter was increased to double its initial value and 20 repeat simulations were carried out over a 10 hour period. The results show the effect on the mean values of NatP, MisP (including MisP bound to Hsps and the proteasome), free pools of Hsp70 and free pools of Hsp90. The arrows show the direction of change, the numbers indicate the proportional change in the species value when the parameter value is doubled (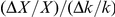), where the denominator is equal to one, if the parameter value *k* is double). Effects which are greater than 10% are indicated in boldface.

#### 2. Parameter scan for model under conditions of high stress

We also looked at the effect of parameters under conditions of high stress by doing a parameter scan on the model with increasing ROS levels. As this model does not produce a steady state until death occurs, we looked at the effect of a two-fold increase in the parameter value on the time to cell death which the deterministic model predicts to take place at 38.8 hours. (Note that this does not equal the median time to cell death in the stochastic model as mentioned previously). The parameters which have the greatest effect on the time to cell death are *k_p38death_*, *k_Jnkdeath_*, *k_p38act_* and *k_remROS_* (Table 5). The first three of these parameters decrease the time to cell death by more than 20% (i.e death occurs before 31.3 hours), whereas a two-fold increase in *k_remROS_* delays cell death by about 40% (death occurs at about 53.8 hours). Table 5 shows all the parameters which have more than a 1% effect on the time to cell death. These parameters are either involved in ROS turnover, cell death pathways, or protein misfolding and aggregation.

**Table 5:** Results of parameter scan for deterministic model with increasing ROS over time.

Size of effect	Parameters which delay cell death	Predicted time of cell death (hours)	Parameters which hasten cell death	Predicted time of cell death (hours)
30–40%	*k_remROS_*	53.8		
20–30%			*k_p38death_*	30.4
20–30%			*k_Jnkdeath_*	30.5
20–30%			*k_p38act_*	30.1
10–20%	*k_dephosJnkMkp1_*	44.9	*k_phosJnk_*	33.1
10–20%	*k_dephosp38Mkp1_*	45.0	*k_phosp38_*	32.9
5–10%			*k_misfold_*	36.7
5–10%			*k_genROS_*	36.6
1–5%	*k_binMisPProt_*	39.3	*k_agg_*	38.5
1–5%			*k_genROSp38_*	38.4
1–5%			*k_PIdeath_*	38.6

The results show the effect of a two-fold increase in the parameter value on time to cell death (predicted time to cell death for default parameters  = 38.8 hours).

#### 3. Global parameter scan for model under normal conditions

In order to further examine the sensitivity of the model to changes in parameter values a global parameter scan in which each parameter was varied simultaneously was carried out using the deterministic model. As the model contains 60 parameters, we generated 50 sets of parameters in which each parameter was randomly assigned a value between half and double its initial value. Each model was run in COPASI and the results were analysed and plotted in R. The full results are given in the supplementary information (Text S1 and Table S1) and summarised in Table 6. This shows that there are two main types of effect, either increases in Hsps with an increase in NatP and decrease in total MisP or decreases in Hsps with a decrease in NatP and increase in total MisP. The first group is normally associated with a decrease in ROS and the second with an increase in ROS. Closer examination of the parameter sets that produced these results reveal that in the sets with large changes in the variables, there are large changes in some of the more sensitive parameters in the model which are given in Table 4. For example, the sets in which levels of NatP and Hsps decrease and MisP increases have larger values for the rate of misfolding, and the amount of MisP increases further if the parameter for ROS generation is increased and/or the parameter for ROS removal is decreased (compare parameter Set 1 and Set 6 in the supplementary information Text S1).

**Table 6:** Summary of global parameter scan results.

	ROS ↓	ROS ↑	ROS ↔ (<5% change)
NatP ↑, Hsps ↑, MisP ↓	19	2	2
NatP ↓, Hsps ↓, MisP ↑	3	15	1
NatP ↔, Hsps ↔, MisP ↑	1	1	0
NatP ↔, Hsps ↔, MisP ↓	1	1	0
Less than 10% change in NatP, Hsps and MisP	4	0	0

## Discussion

Neurodegeneration is an age-related disorder which is characterised by the loss of a subset of neurons in specific regions of the brain and the accumulation of aggregated protein. The proteins involved and the regions of the brain affected are dependent on the particular disease which suggests that stochastic effects may play an important role. Despite individual differences, there seems to be common mechanisms involved which involve loss of protein homeostasis due to cellular systems for removing and repairing damage being overwhelmed. We built a stochastic model to investigate the processes involved, focussing on the role of Hsp70 and Hsp90 in preventing both protein aggregation and cell death. We extended the chaperone model of Proctor *et al.* [12] to include the molecular chaperone Hsp70, clients of Hsp70 and Hsp90 and apoptotic pathways. We used the model to examine the effects of transient moderate stress and the effects of a gradual increase in stress over time to reflect the ageing process. The model predicts that protein homeostasis can be maintained during short periods of stress due to the abundance of chaperones in cells and the fact that misfolded protein competes with clients for Hsps when free pools become diminished. So upregulation of Hsps is not necessary for short periods of stress. This was an unexpected result as we had predicted that chaperones would be upregulated after transient moderate stress (Figure 2). However, there are large pools of free Hsps under normal conditions making upregulation unnecessary unless the stress is severe or prolonged.

Under very long periods of stress, the chaperone system eventually becomes overwhelmed as the upregulation of Hsps cannot keep pace with the increasing demand of protein misfolding. This is consistent with experimental data described in the Introduction, which show that the capacity of chaperone systems can be exceeded under conditions of high external stress [22], [23]. As the chaperone system becomes overwhelmed there is a decline in free pools of the anti-apoptotic Hsp70 and so the probability of cell death pathways being activated increases leading to neuronal loss.

Since neuronal loss has many adverse effects on brain function, a potential therapeutic target is to inhibit cell death pathways. This could be achieved by the use of JNK or p38 inhibitors. However, the model shows that inhibiting either or both of these pathways may delay cell death but that cell death will still ultimately occur via other apoptotic pathways. For example, p53 which is normally rapidly turned over by the proteasome may start to accumulate if aggregated protein inhibits proteasomal function. Indeed, the model predicts that preventing cell death does not stop the aggregation process and eventually cells will die due to proteasome inhibition. The model predicts that if aggregates are sequestered into inclusion bodies, then cell death is less likely to occur although it is necessary for inclusions to form before aggregates start binding to the proteasome. However, it is likely, that high levels of inclusions will also result in cell death [26], and so even if it is possible to manipulate this pathway, it may not be beneficial. Therefore, we suggest that any intervention to inhibit cell death pathways also needs to be able to reduce protein misfolding and aggregation either by enhancing clearance systems or by reducing levels of stress within cells. Our models indicate that the latter strategy may be particularly beneficial.

Although it is generally accepted that an increase in ROS-mediated macromolecular damage contributes to ageing, a causal relationship between ROS and proteotoxicity is still controversial. It has been suggested that an increase in oxidative damage with age could be due to an age-related increase in ROS production [27], although other possibilities include a decline in the efficiency of antioxidant systems or an increase in damaged mitochondria which produce more ROS. An increase in ROS with time can be modelled by either increasing the rate of ROS production or decreasing the rate of ROS removal over time. The outcome in the model would be the same and we chose to allow the level of ROS to slowly increase linearly with time. It might be preferable to include mitochondria in the model and then to use ROS generation from the mitochondria as an input instead of the reaction for ROS generation. This would add further complexity to the model but would provide a more complete picture of the processes involved. In addition a constant pool of ATP could be replaced by reactions of ATP production and consumption where the production rate depends on the state of the mitochondria, e.g. damaged mitochondria produce less ATP. This would be an interesting future development of the current model. Other perturbations which could be carried out in the current model include examining a lowered efficiency of Hsf1 transcription or a decline in proteasome efficiency with age.

The model developed here makes several predictions that are potentially testable using cell culture and animal models. In general there is relatively little information in the chaperone literature on the extent to which various chaperone pools are engaged in the folding process [28]. Kamal *et al*. have compared the extent to which Hsp90 is complexed with cochaperones in normal and tumor cells, using this as a marker of Hsp90 engagement with client proteins [29]. These experiments suggested that only a small fraction of Hsp90 in normal cells is actively engaged in protein folding. It would be interesting to perform similar analyses on cells in which ROS levels have been manipulated experimentally. This might give quantitative experimental data on levels of total and free Hsp90 which could then be compared with model predictions.

Many potential therapeutic strategies for neurodegenerative disorders are targeting JNK and p38. However, our model predictions suggest that although these therapies may be useful in delaying the onset of disease pathology, they will not prevent the early stages of disease or its progression. For example the model predicts that inhibition of p38 and JNK will delay cell death but that eventually inhibition of proteasomes by aggregated protein will cause death in a significant proportion of cells. Previous studies on the role of p38 and JNK have only assessed apoptosis at a short time interval after initiation of protein misfolding in cells and thus are compatible with this model. Assessing apoptosis at longer intervals after initiating protein misfolding would provide a test of this model prediction. This experiment could be performed in the absence and presence of constitutively-active Hsf1 to determine whether a combination of stress-activated protein kinase inhibition and enhancement of chaperone activity is more effective at inhibiting apoptosis than either treatment alone. This would give insights into whether a combination of therapeutic strategies such as increasing chaperone activity to reduce protein aggregation and inhibiting p38 and JNK pathways would be beneficial. Other therapies that could be tested in combination with JNK or p38 inhibitors are the use of antioxidants to reduce protein damage and aggregation, or Hsp90 inhibitors which would reduce aggregation by increasing chaperone activity and reducing levels of mutant tau and may also have effects on apoptosis by reducing the level GSK3β. As recently suggested by Lindner & Demarez, merging predictive modelling and quantitative experimentation will bring new breakthroughs in our understanding of the ageing process [30].

Although the model is quite complex, it is still a simplification of the cellular system and it may be desirable to add in further detail in order to determine the main pathways involved in maintaining protein homeostasis. The level of detail chosen in the current model was based on the current knowledge of the system and the relevance to ageing especially in the context of neurodegeneration. The model was built in SBML, a computer readable format for representing computer models of biological processes. SBML models can be used by an extensive variety of software tools without the need for recoding the model which means that models can be easily shared and adapted by other users. Therefore, the model can easily be adapted by other users and it will be straight forward to add further detail as new information about the system emerges. It is also possible to remove some of the current detail. For example the reactions involving Akt could be removed and then we would assume that Akt is one of the generic Hsp90 clients. In fact removing these reactions did not affect the qualitative behaviour of the model but we chose to include them as it gave insights into the effect of Hsp90 inhibition on Hsp90 clients. Since Akt levels declined when Hsp90 is inhibited we would expect that other clients such as GSK3β and mutant tau would also decline. Therefore we predict that inhibition of Hsp90 might lead to less cell death even though the current model does not show this. On the other hand Akt inhibits GSK3β activity and so the effect of Hsp90 inhibition on GSK3β is not entirely clear and requires further investigation. An important future extension to the model is the addition of GSK3β and tau. Both proteins are linked to Akt since Akt phosphorylates GSK3β and it has been shown that Akt plays an important role in regulating tau degradation [31]. As well as the chaperone system, the ubiquitin-proteasome system plays an important role by removing damaged and misfolded proteins. Our model includes protein degradation but does not have detail of the ubiquitination steps. A model of the ubiquitin-proteasome has also been developed by Proctor *et al.* [32] and since this model was also coded in SBML, it will be possible to link the models together in order to explore the interactions between the chaperone and proteasome pathways in more detail. Hsp70 and Hsp90 are both degraded via CHIP and it may be important to add further detail of these degradation pathways. The molecular chaperone Hsp27 (P04792) is also important in preventing protein aggregation and is also anti-apoptotic. It predominantly exists as oligomers which have chaperone activity, but in stressed conditions oligomers are disrupted by phosphorylation of Hsp27 to form monomers and dimers. One of the kinases involved is p38 which as previously mentioned is itself activated by stress. Hsp27 oligomers also have a role in modulating ROS levels by induction of glutathione levels. A separate model of Hsp27 is currently being developed and this will be incorporated into the model of Hsp70 and Hsp90 to provide a more complete model of the chaperone system.

Our current model includes generic pools of Hsp70 and Hsp90 clients and also some specific clients such as Akt. As already mentioned, another client of Hsp90 which plays an important role in neurodegeneration is (GSK3β) which is involved in phosphorylation of tau, production of amyloid beta (Aβ (P05067)), and modulates apoptotic pathways (reviewed in [33], [34]). It also interacts with p53 and activity of both proteins is increased as a result of this interaction [35]. A stochastic model of the role of GSK3β and p53 has been developed [36] and so it will be of great interest to add GSK3β to the chaperone model and then link the models together to examine the effect of Hsp90 inhibition on the aggregation kinetics of tau and Aβ. We assumed that JNK and p38 were both directly activated by ROS. Recent data suggests that the catalytic Cys residue Mkp1 is oxidised and so deactivated by ROS [37]. Therefore we also assumed that Mkp1 deactivation depends on ROS levels and so an increase in ROS may lead to reduced dephosphorylation of JNK and p38. However, ROS also induces upregulation of Hsp70 which results in more active Mkp1. Our model predicts that increasing ROS leads to inactivation of Mkp1 as the increased pools of Hsp70 are required by the increased levels of misfolded protein.

Our model uncovers three main principles about the system. The first is that oxidative stress is the main trigger for the loss in protein homeostasis and so reducing stress is the key to preventing the initiation of the disease process. The second principle is that the ability of the cell to deal with stress via the chaperone and degradation pathways is important especially over long time scales as misfolded and aggregated protein will accumulate and then disease progression is difficult to halt. Finally, inhibiting cell death pathways allows longer cell survival but cannot prevent the aggregation process which will also ultimately lead to cell death.

To summarise, we have developed a mechanistic model of the chaperone system to investigate how loss of protein homeostasis may lead to protein aggregation and cell death, both characteristics of neurodegeneration. We mainly used stochastic simulation so that cellular variability can be examined and the relationship between protein aggregation and cell death can be explored. The model made several predictions and the next stage will be to test these experimentally using cell culture or animal models. One important prediction is that inhibiting either the JNK or p38 cell death pathway may delay cell death but does not stop the aggregation process so that eventually cells die due to aggregated protein inhibiting proteasomal function. In addition to the chaperone system modelled here, the ubiquitin-proteasome system, lysosomal pathways, and mitochondria are involved in age-related neurodegeneration. Mathematical models of these other cellular mechanisms are currently being developed by the authors and will provide the building blocks of an integrative model. This will increase our understanding about how different processes interact to produce systemic outcomes.

## Methods

### Building the stochastic model

We modified our previous model of Hsp90 [12] which modelled the role of Hsp90 in maintaining protein homeostasis. The earlier model was encoded in the SBML [13] and so is easily modified. As in our previous model, we assume that there is a pool of proteins which are in their native conformation (NatP) but that these proteins are continuously subjected to damage which results in misfolding. There are three outcomes for misfolded proteins (MisP). Firstly, the molecular chaperones Hsp70 and Hsp90 bind to MisP to prevent their aggregation and an attempt is made to refold the proteins in an ATP-dependent reaction. Secondly, Hsp70 or Hsp90 will transport misfolded protein to the ubiquitin-proteasome system for degradation, although we do not include details of the ubiquitination steps as previously modelled [32]. Lastly the misfolded proteins may aggregate to form a small aggregate (AggP). Further misfolded protein may then bind to a small aggregate to form a larger aggregate (Seq AggP) which we will also refer to as an inclusion body. Small aggregates may bind to the proteasome and inhibit proteasomal function (AggP_Proteasome) and may also increase the generation of ROS. We assume that SeqAggP does not interfere with the cellular machinery and is a means to isolating the toxic aggregates. We also assume that Hsp70 and Hsp90 may be damaged by ROS and that damaged forms lose their chaperone activity and may form small aggregates or be sequestered into inclusion bodies in a similar way to misfolded protein.

Hsp70 and Hsp90 both have many client proteins which bind with high affinity to form complexes, but are constantly undergoing cycles of assembly and disassembly. We represent these clients with generic pools named Hsp70Clients and Hsp90Clients.

We also include the Hsp90 client Akt since this kinase is important in many pathways and so provides a link to other models. In addition, there is experimental data on the half-life of Akt and how this is affected by Hsp90 inhibition providing useful information for the parameters involved in Akt turnover. Hsp90 binds Heat Shock transcription Factor-1 (Hsf1) which keeps Hsf1 in its inactive monomeric state which we include as a separate species. Under conditions of stress, an increase in misfolded protein sequesters Hsf1 from Hsp90 and the increase in the pool of unbound Hsf1 enables dimerisation and trimerisation to take place. Hsf1 trimers are then phosphorylated by a protein kinase (PKC, (IPR015745)) which makes Hsf1 transcriptionally active. Hsf1 trimers can bind to the Heat Shock Element (HSE) in either their phosphorylated or un-phosphorylated state but only the former binding leads to transcription of heat shock proteins. We also include basal synthesis and degradation of Hsp70 and Hsp90.

Among the clients for Hsp70 is the currently unknown phosphatase responsible for dephosphorylating Hsf1 trimers, (which we name PPX). We assume that under normal conditions PPX is in complex with Hsp70 and so Hsf1 trimers are in their unphosphorylated state. Hsp70 also activates the phosphatase Mkp1 which is responsible for dephosphorylating JNK and p38 [38]. When free pools of Hsp70 are high, Mkp1 is in its active state, but if Hsp70 pools are depleted, Mkp1 becomes inactivated and so JNK and p38 are more likely to be in a phosphorylated state. Under conditions of stress misfolded protein binds preferentially to Hsp70 leading to an increase in phosphorylated JNK and Hsf1 trimers. This leads to an increase in the transcription of heat shock genes. If JNK remains in a phosphorylated state, it will contribute to apoptotic signalling. However, if Hsp70 levels increase due to the transcriptional activity of Hsf1, then JNK will be de-phosphorylated before apoptotic signalling takes place. We assume that JNK and p38 are both phosphorylated in response to signalling via ROS, so if ROS levels are high as a result of an increase in stress, the rate of phosphorylation increases.

A list of species and reactions are shown in Tables 7 and S2 respectively. The model was encoded in SBML shorthand and converted to full SBML [39]. It was then imported into the BASIS modelling system [40] and simulations were run using a stochastic simulator based on the Gillespie algorithm [41]. To keep the model simple, we have not included detail of the apoptotic pathways. Models of apoptosis will be developed separately and then linked to this model if required. Instead, we assume that a cell will die if any of the following three conditions occur. The first two conditions involve activation of either JNK or p38, where we assume that the probability of cell death increases as pools of activated JNK or p38 increase. The third condition is inhibition of the proteasome where we assume that as the pool of AggP_Proteasome increases (and hence the available pool of proteasomes decreases) the probability of cell death increases. These conditions are modelled by reactions in SBML and the details are given in Table S2. Note that since the model is stochastic, cell death may occur even when pools of activated JNK or p38 are fairly low but with a very low probability. When a death reaction occurs, a dummy species is set to one, so that cell deaths can be counted according to type. A dummy parameter *kalive* is present in all the reactions and is initially set to one. When a cell death occurs, the parameter is set to zero so that no more reactions can take place in the simulation. This is achieved by using SBML event structures (Table 2). The SBML code is available from the BASIS website [42], and a fully annotated version can be obtained from the Biomodels database (MODEL1005280000) [43], [44]. The code is also available in Code S1 in the supplementary materials. Simulation results were analysed and plotted using R.

**Table 7:** List of Species.

Species description	Species Name	Database term	Initial Amount
Native protein	NatP	CHEBI:36080	17600
Misfolded protein	MisP	CHEBI:36080	0
Aggregated protein	AggP	CHEBI:36080	0
MisP bound to Hsp70	Hsp70_MisP	IPR001023, CHEBI:36080	470
MisP bound to Hsp90	Hsp90_MisP	IPR001404, CHEBI:36080	410
Hsp70	Hsp70	IPR001023	1400
Hsp90	Hsp90	IPR001404	1850
Damaged Hsp70	Hsp70_dam	IPR001023	0
Damaged Hsp90	Hsp90_dam	IPR001404	0
Hsp70 bound to proteasome	Hsp70_Proteasome	IPR001023, GO:0000502	0
Hsp90 bound to proteasome	Hsp90_Proteasome	IPR001404 GO:0000502	0
Hsf1	Hsf1	Q00613	5
Hsf1 bound to Hsp90	Hsf1_Hsp90	Q00613, IPR001404	95
Hsf1 dimers	Hsf1_Hsf1	Q00613	0
Hsf1 trimers	Hsf1_Hsf1_Hsf1	Q00613	0
Phosphorylated Hsf1 trimers	Hsf1_Hsf1_Hsf1_P	Q00613	0
Hsp70 heat shock element	HSEHsp70	SBO:0000369	2
Hsp90 heat shock element	HSEHsp90	SBO:0000369	2
Phosphorylated Hsf1 bound to HSEHsp70	HSEHsp70_Hsf1_Hsf1_Hsf1_P	SBO:0000369, Q00613	0
Unphosphorylated Hsf1 bound to HSEHsp70	HSEHsp70_Hsf1_Hsf1_Hsf1	SBO:0000369, Q00613	0
Phosphorylated Hsf1 bound to HSEHsp90	HSEHsp90_Hsf1_Hsf1_Hsf1_P	SBO:0000369, Q00613	0
Unphosphorylated Hsf1 bound to HSEHsp90	HSEHsp90_Hsf1_Hsf1_Hsf1	SBO:0000369, Q00613	0
Hsp70 client proteins	Hsp70Client	CHEBI:36080	490
Hsp90 client proteins	Hsp90Client	CHEBI:36080	590
Hsp70 bound to clients	Hsp70_Hsp70Client	IPR001023, CHEBI:36080	10
Hsp90 bound to clients	Hsp90_Hsp90Client	IPR001404, CHEBI:36080	10
Akt (protein kinase B)	Akt	P31749	340
Akt bound to Hsp90	Akt_Hsp90	P31749, IPR001404	30
Carboxy terminus of Hsp70-interacting protein	CHIP	Q9UNE7	255
Akt bound to CHIP/Hsp90 complex	Akt_CHIP_Hsp90	P31749, Q9UNE7, IPR001404	80
Akt bound to proteasome	Akt_Proteasome	P31749, GO:0000502	0
Proteasome	Proteasome	GO:0000502	500
MisP bound to proteasome	MisP_Proteasome	CHEBI:36080,GO:0000502	0
Aggregated protein bound to proteasome	AggP_Proteasome	CHEBI:36080, GO:0000502	0
Sequestered aggregated protein	SeqAggP	CHEBI:36080	0
Phosphatase Mkp1 (DUSP1)	Mkp1	P28562	0
Phosphorylated Mkp1	Mkp1_P	P28562	100
Mkp1 bound to proteasome	Mkp1_Proteasome	P28562, GO:0000502	0
Phosphatase for Hsf1	Ppx	GO:0008287	0
PPX bound to Hsp70	Hsp70_Ppx	IPR001023, GO:0008287	100
JNK (MAPK8)	Jnk	P45983	100
Phosphorylated JNK	Jnk_P	P45983	0
p38MAPK (MAPK14)	p38	Q16539	100
Phosphorylated p38MAPK	p38_P	Q16539	0
Protein kinase C	Pkc	IPR015745	100
Reactive oxygen species	ROS	CHEBI:26523	10
Adenosine triphosphate	ATP	CHEBI:15422	10000
Adenosine diphosphate	ADP	CHEBI:16761	1000

IPR: InterPro [48].

GO: Gene ontology [49].

CHEBI: Chemical Entities of Biological Interest database [50].

P and Q: Uniprot [51].

SBO: Systems Biology Ontology [52].

### Use of stochastic and deterministic models

Stochastic effects are very important in this model under conditions of moderate or high stress. In particular the timing of when the aggregation process starts, the destination of aggregates (inhibition of the proteasome or sequestered into inclusion bodies), the levels of ROS and the timing of cell death show large variations between simulation runs. However, stochastic simulations are very computer intensive and for conditions of high stress a set of 100 repeat runs takes about one week if run on a PC or about 24 hours if run on the BASIS cluster. Therefore it is not feasible to use a stochastic model to carry out a full parameter scan. To overcome this problem we also developed a deterministic model and ran simulations in CellDesigner and COPASI. We used the same initial conditions and parameter values for the deterministic model so that direct comparisons could be made. The rate laws for dimerisation reactions were adjusted, for example *k_dimerHsf1_*<#Hsf1><#Hsf1-1>/2.0 was changed to *k_dimerHsf1_*Hsf1^2^.

### Initial amounts of species

Proteins are present in very large numbers in cells. For example, experimental values of the number of p38 molecules per cell has been estimated as 10^6^ molecules per cell [45]. As we are using stochastic simulation, it is not practical to have starting values in this order of magnitude due to the amount of time that would be required to carry out even a single simulation. Therefore it is necessary to scale down the values of model species. The motivation for the model was to examine the qualitative behaviour of the system and as information on initial amounts for the majority of species is not available we have assumed that most species are present in relatively similar abundances but that Hsps are an order of magnitude more abundant than kinases, and the generic pool of NatP was set to be two orders of magniture more abundant as this represents many different proteins. We used a constant value of 10^4^ molecules/cell for ATP since our model includes only a small fraction of the reactions requiring ATP and it does not seem appropriate to assume that ATP levels would be rate limiting in this model. We assumed that the cell volume is equal to one so that all initial amounts represent the number of molecules per cell and so we used the same initial values in the deterministic simulations.

### Parameter values

One of the most difficult parts in the model building process is finding values for all the parameters as kinetic data is often not available. Where kinetic data is available, it is often from *in vitro* systems which may not accurately reflect the *in vivo* kinetics. Also kinetic parameters are often dependent on cell type. However, even if exact values are not easy to obtain, it is often satisfactory to know the relative time scales of the reactions. For example, kinase/phosphatase reactions are very fast reactions occurring over timescales that range from fraction of a second to seconds, whereas protein synthesis takes several minutes. Degradation rates are dependent on protein type but fortunately, there is usually information available on protein half-lives which can be used to calculate the degradation rate. Note that the half-life of a protein is dependent on the rate at which the protein is targeted for degradation rather than on the rate of proteasome activity which we assume to be independent of protein type (apart from aggregated protein which we assume is totally resistant to degradation by proteasomes). So we set the rate at which a particular protein binds to the proteasome to be equal to –ln(0.5)/t_0.5_, where t_0.5_ is the protein half-life. As an example we assume that the half-life of Hsp70 is about 30 hours, so that *k_binHsp70Prot_* * Proteasome  = −ln(0.5)/108000s. Since the pool of proteasomes is about 500, then *k_binHsp70Prot_*  = 1.2×10^−8^. We then set the basal synthesis rate, so that total Hsp70 pools remain constant under normal conditions. We set the parameters for aggregation to be very low, since it has been shown that there is a very long lag phase before aggregates start to form and also we would not expect aggregation to take place under normal conditions. Once we had chosen a set of parameters, we then simulated the model under normal conditions and checked that all species remained fairly constant over time. As the model is stochastic we would expect variations in protein levels over time but there should be no obvious trend of an increase or decrease in these levels. If such a trend was observed, we made the necessary adjustments to the parameter values. We also checked the model by doing 100 repeat simulations and plotting the mean values, and in addition ran the model in a deterministic simulator using CellDesigner [46]. We carried out parameter scans for all the parameter values using COPASI [25] (see Results section).

### Model validation

After choosing the parameters for normal conditions, we then validated the model against experimental data for the heat shock response. We do not have temperature as a variable in our model, however the transient increase in ROS levels has a similar effect to increasing the temperature for a specified time period. Kline & Morimoto et al measured the dynamics of phosphorylated Hsf1, binding of Hsf1 to HSE and the transcription rate of Hsp70 after heat-shock of HeLa cells at 42°C for 250 minutes [47]. They observed a rapid activation of all three variables between 0 and 35 minutes after the stress with a attenuation back to basal levels over the next ∼200 minutes. Note that the attenuation phase begins during the continued heat-shock exposure. We examined the dynamics of the same variables from our simulation output under conditions of ROS increase for 4 hours. The model predicts that there is a rapid activation of all three variables which begins about 1 minute after the start of the stress response, reaches a maximum after about 1–2 hours and then attenuates back to basal levels over the next 2 hours with the attenuation phase beginning during the period of elevated ROS (data not shown). The attenuation phase starts even though ROS is still elevated due to the increase in levels of Hsp70 which will bind to PPX resulting in dephosphorylation of Hsf1 trimers. The qualitative behaviour of our model is in good agreement with the experimental data showing that the model captures the important features of the stress response.

## Supporting Information

Figure S1 Diagram of the model. Numbers on the arrows refer to the reaction numbers in Table S2. Note that some reactions are omitted for clarity where similar reactions occur as noted in the footnotes to Table S2.
(TIF)

Figure S2 Mean of 100 runs for normal conditions. A Native protein, total misfolded protein (includes misfolded bound by Hsps), and reactive oxygen species (ROS). ROS are scaled x100 to allow easier visualisation. B Total Hsp90 (free pools plus all complexes), Free Hsp90 (unbound Hsp90) and Hsp90_MisP (Hsp90 bound to misfolded protein). C Total Hsp70 (free pools plus all complexes), Free Hsp70 (unbound Hsp70) and Hsp70_MisP (Hsp70 bound to misfolded protein).
(TIF)

Figure S3 Deterministic simulation for normal model. A Native protein, total misfolded protein (includes misfolded bound by Hsps), and reactive oxygen species (ROS). ROS are scaled x100 to allow easier visualisation. B Total Hsp90 (free pools plus all complexes), Free Hsp90 (unbound Hsp90) and Hsp90_MisP (Hsp90 bound to misfolded protein). C Total Hsp70 (free pools plus all complexes), Free Hsp70 (unbound Hsp70) and Hsp70_MisP (Hsp70 bound to misfolded protein).
(TIF)

Figure S4 Deterministic simulation for model with transient stress. A Native protein, total misfolded protein (includes misfolded bound by Hsps), and reactive oxygen species (ROS). ROS are scaled x100 to allow easier visualisation. B Total Hsp90 (free pools plus all complexes), Free Hsp90 (unbound Hsp90) and Hsp90_MisP (Hsp90 bound to misfolded protein). C Total Hsp70 (free pools plus all complexes), Free Hsp70 (unbound Hsp70) and Hsp70_MisP (Hsp70 bound to misfolded protein).
(TIF)

Figure S5 Graph to show how ROS levels increase with time. Black line shows rate of ROS production versus time, red and green curves show ROS levels for two stochastic simulations. Horizontal part of curve corresponds to cell death.
(TIF)

Figure S6 Deterministic model for ROS increasing with time. A Native protein, total misfolded protein (includes misfolded bound by Hsps), and reactive oxygen species (ROS). ROS are scaled x100 to allow easier visualisation. B Total Hsp90 (free pools plus all complexes), Free Hsp90 (unbound Hsp90) and Hsp90_MisP (Hsp90 bound to misfolded protein). C Total Hsp70 (free pools plus all complexes), Free Hsp70 (unbound Hsp70) and Hsp70_MisP (Hsp70 bound to misfolded protein).
(TIF)

Figure S7 Effect of varying *k_seqagg_*. The parameter *k_seqagg_* was varied from half to double of its initial value in the deterministic model with ROS increasing with time and inhibition of JNK and p38 death pathways. The parameter scan was carried out in COPASI and the results plotted in R.
(TIF)

Figure S8 Effect of varying *k_upregHsp_*. The parameter *k_upregHsp_* was varied over two orders of magnitude in the deterministic model with ROS increasing with time and inhibition of JNK and p38 death pathways. The scan was carried out in COPASI and the results plotted in R.
(TIF)

Table S1 Results of the global parameter scan. Excel spreadsheet containing full results of 50 randomly chosen parameter sets and the effects on the model predictions. Sheet 1 contains all the parameter values for each set. Sheet 2 shows the percentage difference between each parameter and the default value. Sheet 3 shows the predicted percentage change in mean value for native protein, total misfolded protein, free pools of Hsp70 and Hsp90, and ROS for each parameter set.
(XLS)

Table S2 List of reactions. List of all the reactions in the model including kinetic rate laws and parameter values.
(DOC)

Text S1 Figures for the global parameter scan. Plots showing levels of native protein, misfolded protein and free pools of Hsp70 and Hsp90 for each of the 50 randomly chosen parameter sets. The number below each graph corresponds to the parameter set shown in Table S1.
(PDF)

Code S1 (XML)
