# Supplementary figures and images for: Modelling the Role of the Hsp70/Hsp90 System in the Maintenance of Protein Homeostasis
Source: PLoS One. 2011 Jul 14;6(7):e22038. doi: 10.1371/journal.pone.0022038 (PMC3137010; doi:10.1371/journal.pone.0022038)

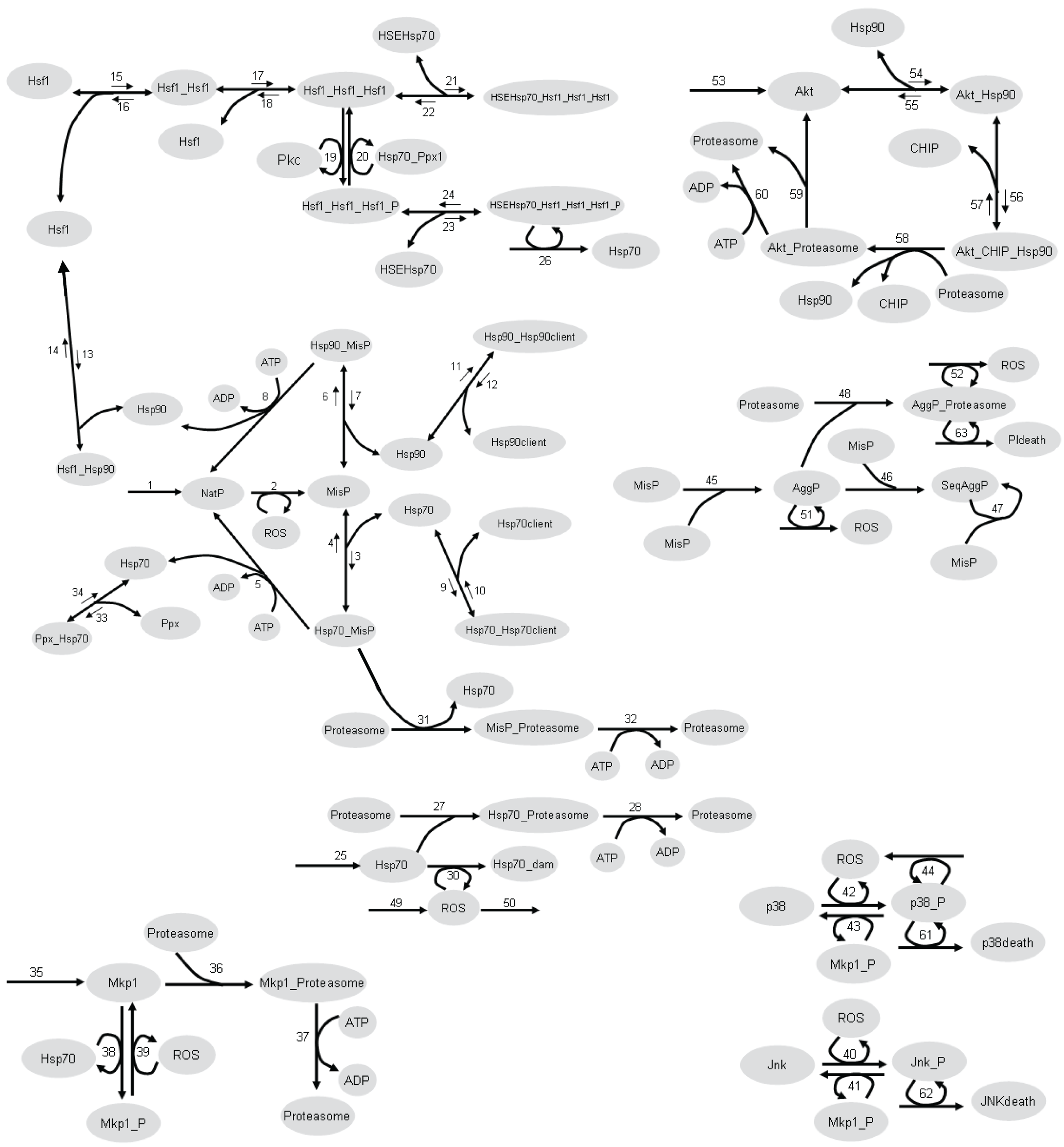

Supplement: Figure S1 — Diagram of the model. Numbers on the arrows refer to the reaction numbers in Table S2. Note that some reactions are omitted for clarity where similar reactions occur as noted in the footnotes to Table S2. (TIF) [file pone.0022038.s001.tif]

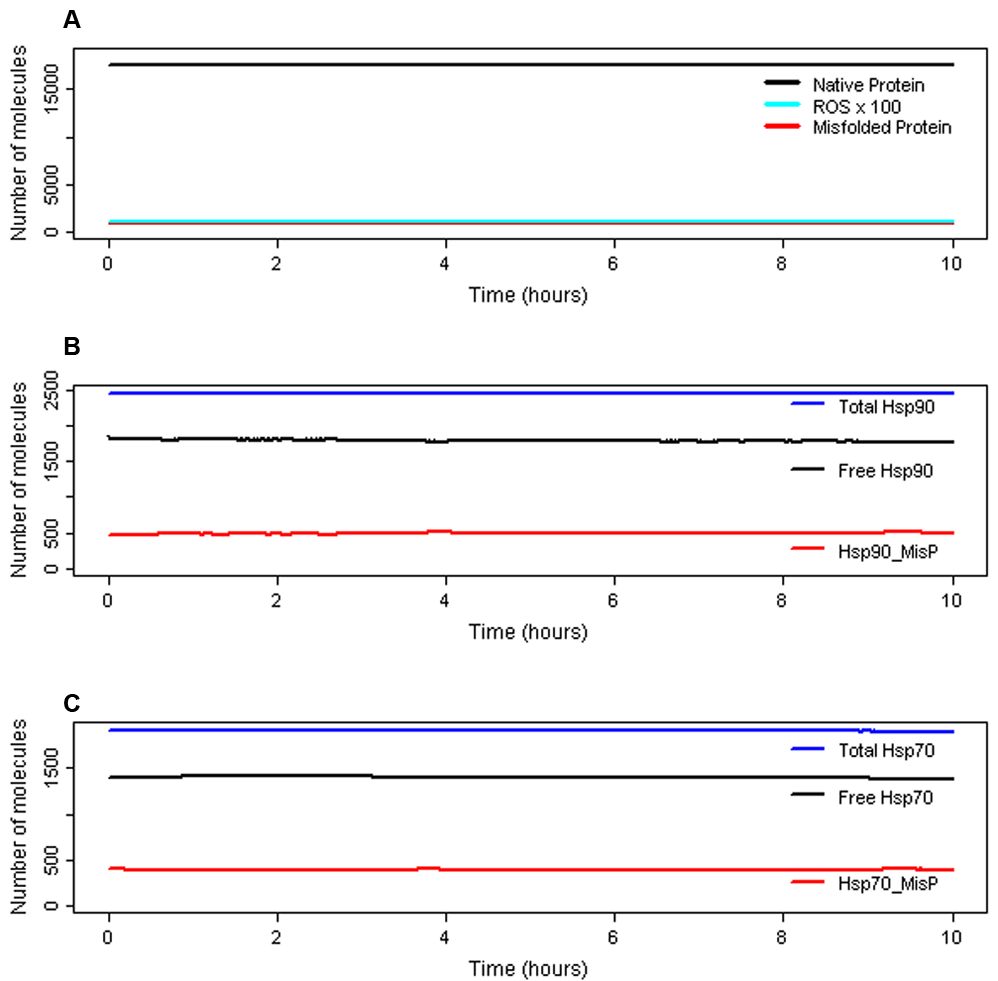

Supplement: Figure S2 — Mean of 100 runs for normal conditions. A Native protein, total misfolded protein (includes misfolded bound by Hsps), and reactive oxygen species (ROS). ROS are scaled x100 to allow easier visualisation. B Total Hsp90 (free pools plus all complexes), Free Hsp90 (unbound Hsp90) and Hsp90_MisP (Hsp90 bound to misfolded protein). C Total Hsp70 (free pools plus all complexes), Free Hsp70 (unbound Hsp70) and Hsp70_MisP (Hsp70 bound to misfolded protein). (TIF) [file pone.0022038.s002.tif]

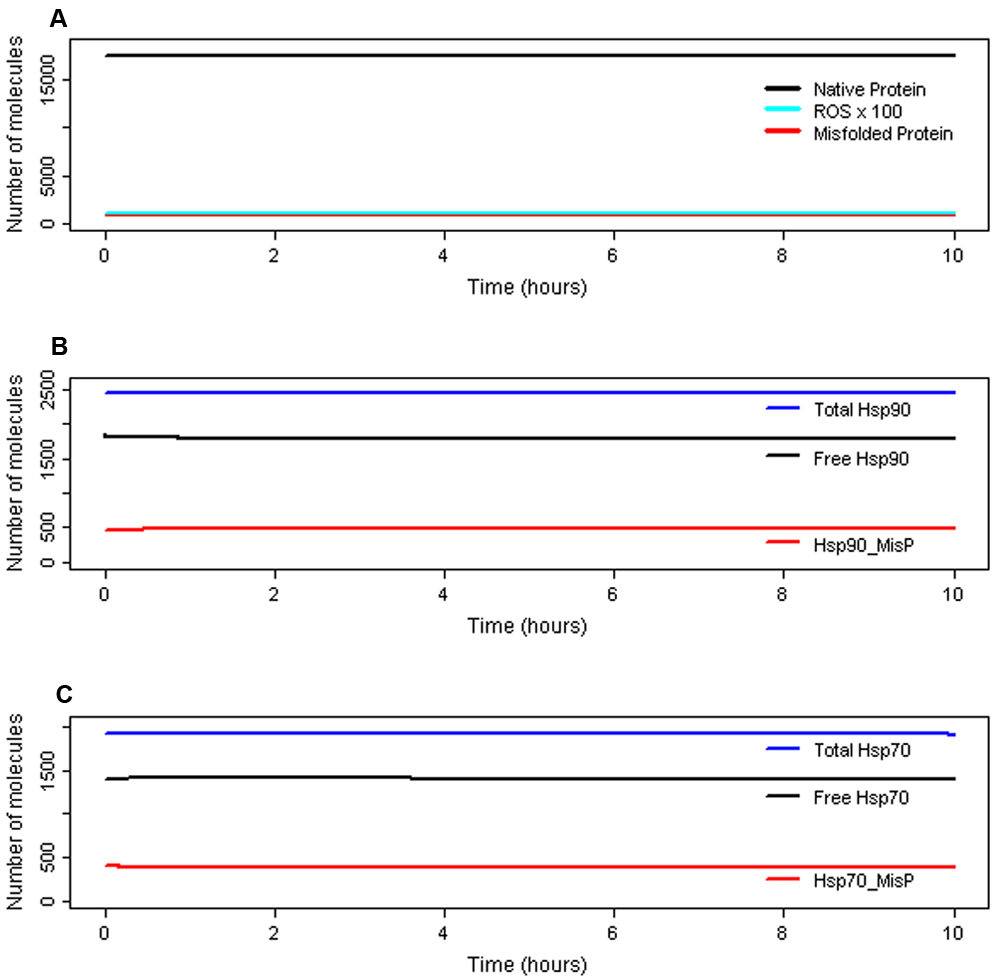

Supplement: Figure S3 — Deterministic simulation for normal model. A Native protein, total misfolded protein (includes misfolded bound by Hsps), and reactive oxygen species (ROS). ROS are scaled x100 to allow easier visualisation. B Total Hsp90 (free pools plus all complexes), Free Hsp90 (unbound Hsp90) and Hsp90_MisP (Hsp90 bound to misfolded protein). C Total Hsp70 (free pools plus all complexes), Free Hsp70 (unbound Hsp70) and Hsp70_MisP (Hsp70 bound to misfolded protein). (TIF) [file pone.0022038.s003.tif]

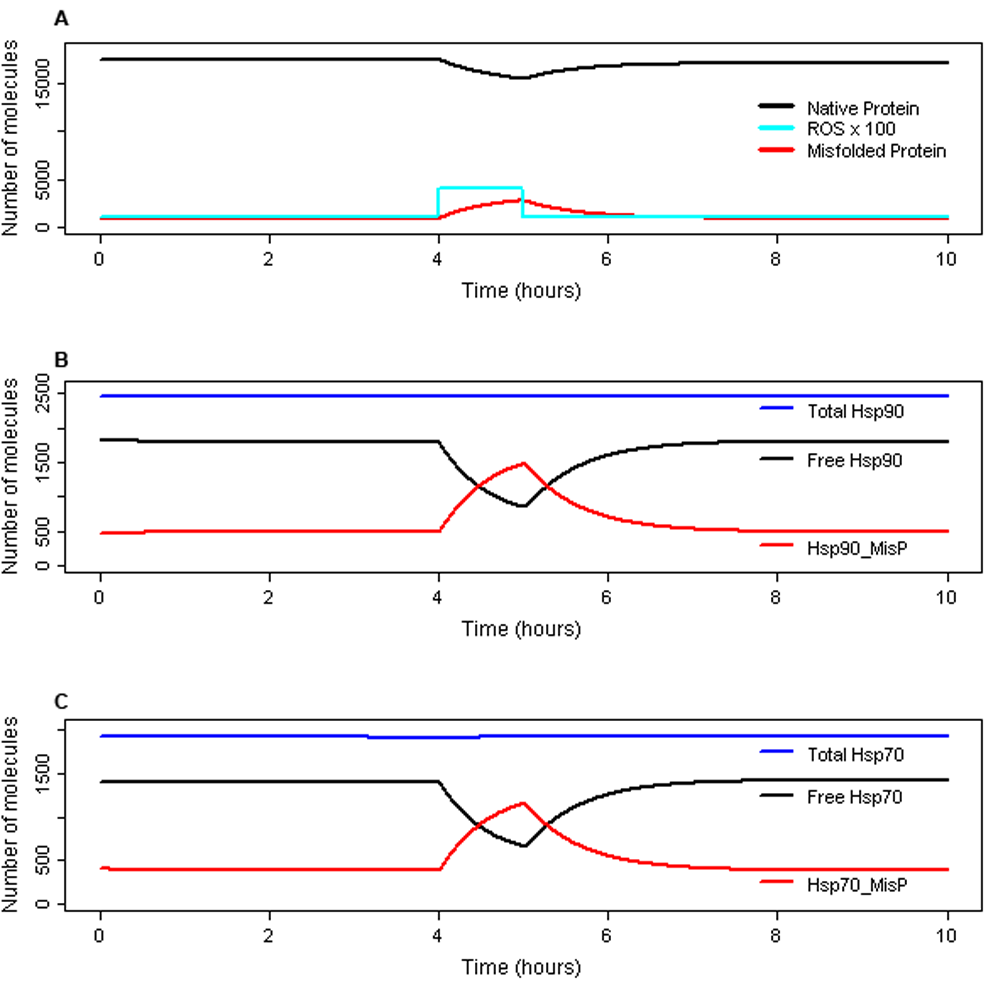

Supplement: Figure S4 — Deterministic simulation for model with transient stress. A Native protein, total misfolded protein (includes misfolded bound by Hsps), and reactive oxygen species (ROS). ROS are scaled x100 to allow easier visualisation. B Total Hsp90 (free pools plus all complexes), Free Hsp90 (unbound Hsp90) and Hsp90_MisP (Hsp90 bound to misfolded protein). C Total Hsp70 (free pools plus all complexes), Free Hsp70 (unbound Hsp70) and Hsp70_MisP (Hsp70 bound to misfolded protein). (TIF) [file pone.0022038.s004.tif]

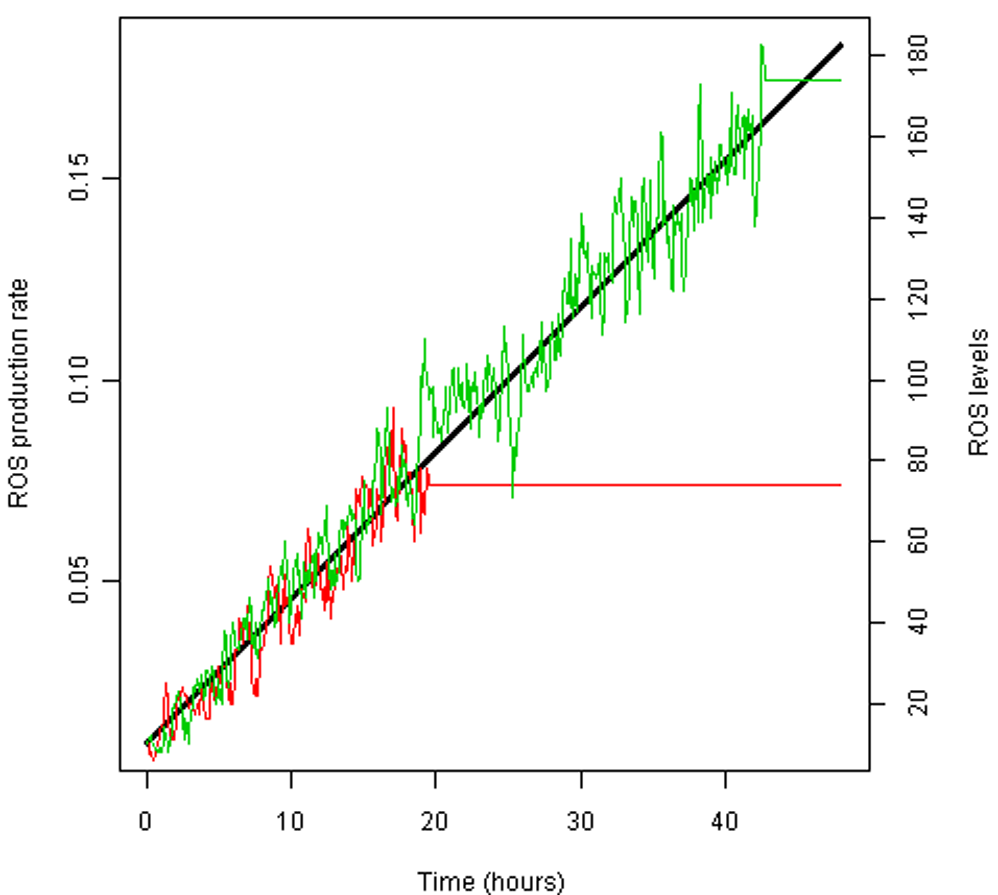

Supplement: Figure S5 — Graph to show how ROS levels increase with time. Black line shows rate of ROS production versus time, red and green curves show ROS levels for two stochastic simulations. Horizontal part of curve corresponds to cell death. (TIF) [file pone.0022038.s005.tif]

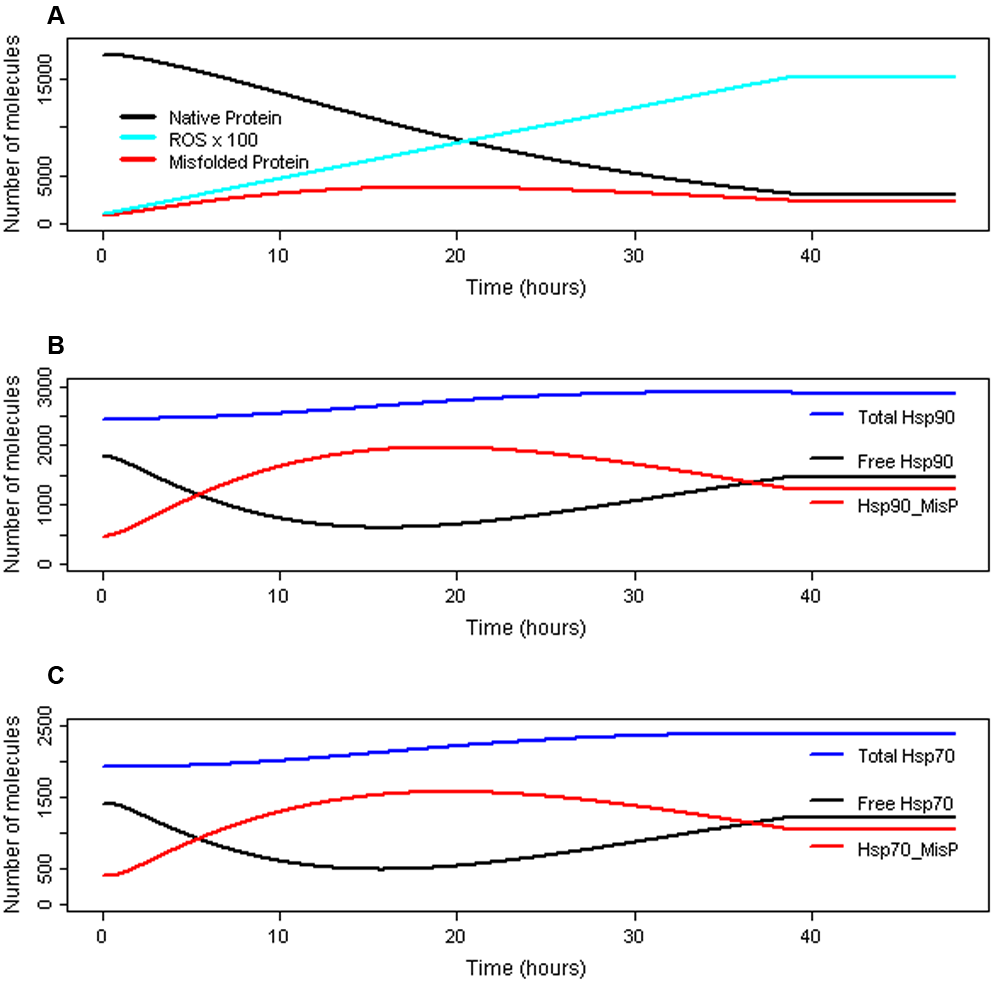

Supplement: Figure S6 — Deterministic model for ROS increasing with time. A Native protein, total misfolded protein (includes misfolded bound by Hsps), and reactive oxygen species (ROS). ROS are scaled x100 to allow easier visualisation. B Total Hsp90 (free pools plus all complexes), Free Hsp90 (unbound Hsp90) and Hsp90_MisP (Hsp90 bound to misfolded protein). C Total Hsp70 (free pools plus all complexes), Free Hsp70 (unbound Hsp70) and Hsp70_MisP (Hsp70 bound to misfolded protein). (TIF) [file pone.0022038.s006.tif]

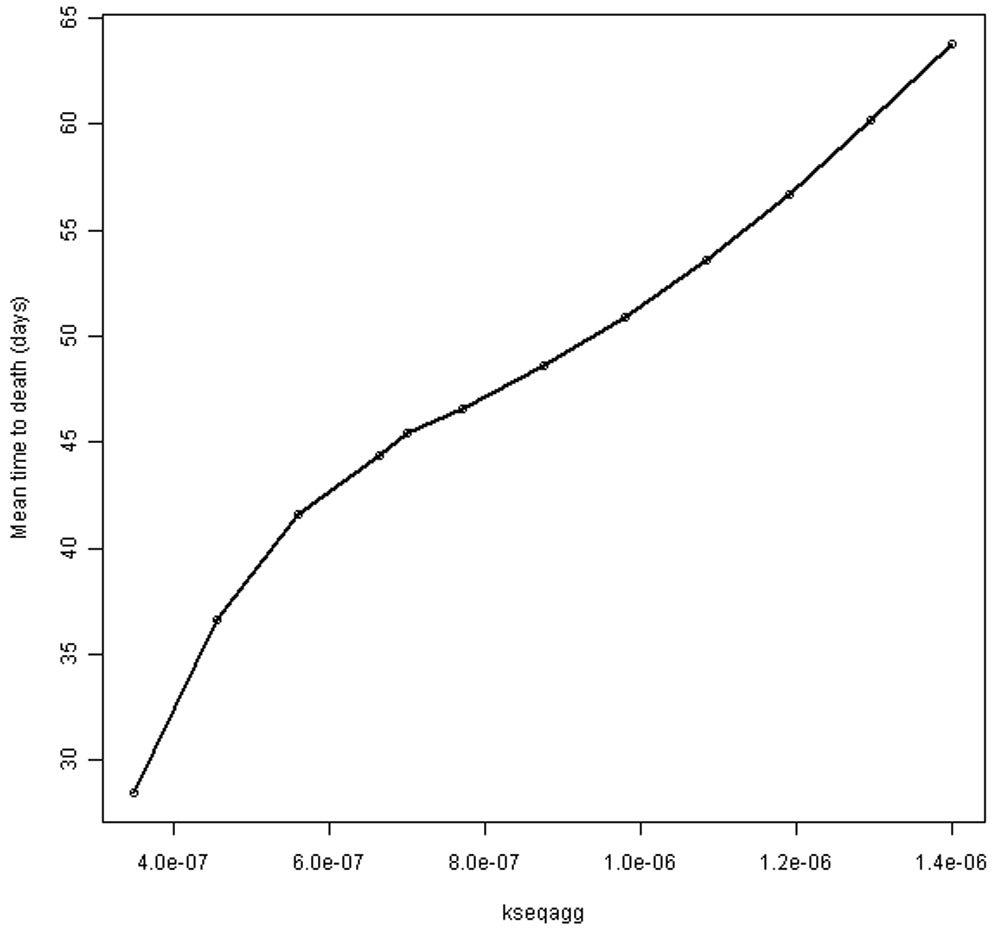

Supplement: Figure S7 — Effect of varying kseqagg. The parameter kseqagg was varied from half to double of its initial value in the deterministic model with ROS increasing with time and inhibition of JNK and p38 death pathways. The parameter scan was carried out in COPASI and the results plotted in R. (TIF) [file pone.0022038.s007.tif]

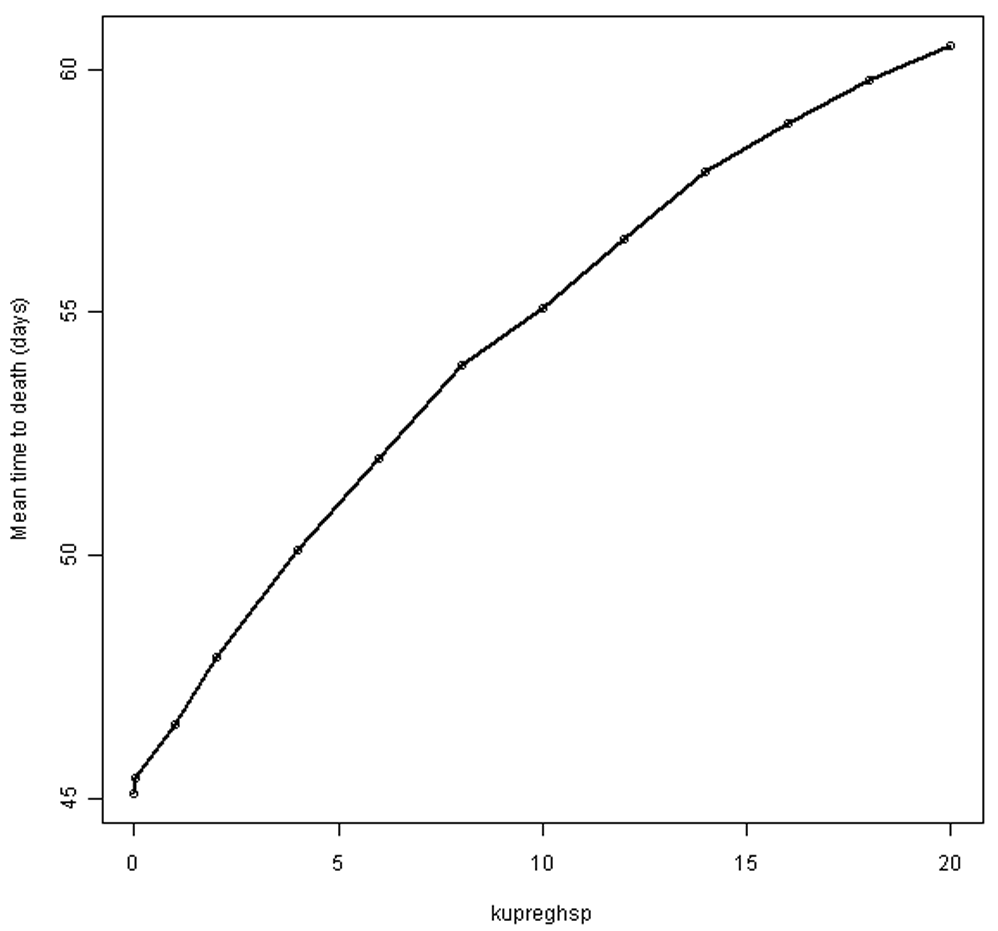

Supplement: Figure S8 — Effect of varying kupregHsp. The parameter kupregHsp was varied over two orders of magnitude in the deterministic model with ROS increasing with time and inhibition of JNK and p38 death pathways. The scan was carried out in COPASI and the results plotted in R. (TIF) [file pone.0022038.s008.tif]
