# Supplementary material for: Modelling the Role of the Hsp70/Hsp90 System in the Maintenance of Protein Homeostasis
Source: PLoS One. 2011 Jul 14;6(7):e22038. doi: 10.1371/journal.pone.0022038 (PMC3137010; doi:10.1371/journal.pone.0022038)
Supplement: Table S2 — List of reactions. List of all the reactions in the model including kinetic rate laws and parameter values. (DOC) [file pone.0022038.s010.doc]

Table S2 List of Reactions

| No. | Reaction | Reactants and Products | Kinetic rate law^a^ | Value |
| --- | --- | --- | --- | --- |
| 1 | Protein synthesis | Source→ NatP | *k_synNatP_* | 4.2E-2 molecule s^-1^ |
| 2 | Misfolding | NatP+ROS→ MisP+ROS | *k_misfold_*<#NatP><#ROS> | 2.0E-6 molecule^-1^s^-1^ |
| 3 | Hsp70/MisP binding | Hsp70+MisP→ Hsp70_MisP | *k_binHspMisP_* <#Hsp70><#MisP> | 8.0E-6 molecule^-1^ s^-1^ |
| 4 | Hsp70/MisP release | Hsp70_MisP→ Hsp70+MisP | *k_relHspMisP_* <#Hsp70_MisP> | 8.0E-5 s^-1^ |
| 5 | Hsp70 refolding | ATP+Hsp70_MisP→ ADP+Hsp70+NatP | *k_refold_* <#Hsp70_MisP>  <#ATP>/(5000+<#ATP>) | 5.5E-4 s^-1^ |
| 6 | Hsp90/MisP binding | Hsp90+MisP→ Hsp90_MisP | *k_binHspMisP_* <#Hsp90><#MisP> | 2.0E-6 molecule^-1^ s^-1^ |
| 7 | Hsp90/MisP release | Hsp90_MisP→ Hsp90+MisP | *k_relHspMisP_* <#Hsp90_MisP> | 8.0E-5 s^-1^ |
| 8 | Hsp90 refolding | ATP+Hsp90_MisP→ ADP+Hsp90+NatP | *k_refold_* <#Hsp90_MisP>  <#ATP>/(5000+<#ATP>) | 5.5E-4 s^-1^ |
| 9 | Hsp70Client binding | Hsp70+Hsp70Client→ Hsp70_Hsp70Client | *k_binHsp70clien_*_t_<#Hsp70>  <#Hsp70Client> | 2.0E-4 molecule^-1^ s^-1^ |
| 10 | Hsp70Client release | Hsp70_Hsp70Client→ Hsp70+Hsp70Client | *k_relHsp70client_*<#Hsp70_Hsp70Client> | 5.0 s^-1^ |
| 11 | Hsp90Client binding | Hsp90+Hsp90Client→ Hsp90_Hsp90Client | *k_binHsp90client_* <#Hsp90>  <#Hsp90Client> | 2.0E-4 molecule^-1^ s^-1^ |
| 12 | Hsp90Client release | Hsp90_Hsp90Client→ Hsp90+Hsp90Client | *k_relHsp90client_* <#Hsp90_Hsp90Client> | 5.0 s^-1^ |
| 13 | Hsp90/Hsf1binding | Hsf1+Hsp90→ Hsf1_Hsp90 | *k_binHsf1Hsp90_*<#Hsf1><#Hsp90> | 2.0E-2 |
| 14 | Hsp90/Hsf1release | Hsf1_Hsp90→ Hsf1+Hsp90 | *k_reHsf1lHsp90_*<#Hsf1_Hsp90> | 0.5 s^-1^ |
| 15 | Hsf1 dimerisation | 2Hsf1→ Hsf1_Hsf1 | *k_dimerHsf1_*<#Hsf1><#Hsf1-1>/2.0 | 8.0E-5 molecule^-1^ s^-1^ |
| 16 | Hsf1 de-dimerisation | Hsf1_Hsf1→2Hsf1 | *k_dedimerHsf1_*<#Hsf1_Hsf1> | 0.5 s^-1^ |
| 17 | Hsf1 trimerisation | Hsf1+Hsf1_Hsf1→ Hsf1_Hsf1_Hs1 | *k_trimerHsf1_*<#Hsf1><#Hsf1_Hsf1> | 1.0E-2 molecule^-1^ s^-1^ |
| 18 | Hsf1 de-trimerisation | Hsf1_Hsf1_Hsf1→ Hsf1+Hsf1_Hsf1 | *k_detrimerHsf1_*<# Hsf1_Hsf1_Hsf1> | 0.5 s^-1^ |
| 19 | Hsf1 trimer phosphorylation | Hsf1_Hsf1_Hsf1+Pkc→ Hsf1_Hsf1_Hsf1_P+Pkc | *k_phosHsf1_*<# Hsf1_Hsf1_Hsf1> <#Pkc> | 3.0E-2 molecule^-1^ s^-1^ |
| 20 | Hsf1 trimer de- phosphorylation | Hsf1_Hsf1_Hsf1_P+Hsp70_Ppx→ Hsf1_Hsf1_Hsf1+Hsp70_Ppx | *k_dephosHsf1_* <#Hsf1_Hsf1_Hsf1_P>  <#Hsp70_Ppx> | 1.0E-2 molecule^-1^ s^-1^ |
| 21 | HSEHsp70/Hsf1 trimer binding^b^ | HSEHsp70+Hsf1_Hsf1_Hsf1→ HSEHsp70_Hsf1_Hsf1_Hsf1 | *k_binHSEHsf1_*<#HSEHsp70> <#Hsf1_Hsf1_Hsf1> | 5.0E-2 molecule^-1^ s^-1^ |
| 22 | HSEHsp70/Hsf1 trimer release^b^ | HSEHsp70_Hsf1_Hsf1_Hsf1→ HSEHsp70+Hsf1_Hsf1_Hsf1 | *k_relHSEHsf1_*  <#HSEHsp70_Hsf1_Hsf1_Hsf1> | 8.0E-2 s^-1^ |
| 23 | HSEHsp70/Hsf1 phospho-trimer binding^b^ | HSEHsp70+ Hsf1_Hsf1_Hsf1_P→ HSEHsp70_Hsf1_Hsf1_Hsf1_P | *k_binHSEPhosTriH_*<#HSEHsp70> <#Hsf1_Hsf1_Hsf1_P> | 0.1 molecule^-1^ s^-1^ |
| 24 | HSEHsp70/Hsf1 phospho-trimer release^b^ | HSEHsp70_Hsf1_Hsf1_Hsf1_P→ HSEHsp70+Hsf1_Hsf1_Hsf1_P | *k_relHSEPhosTriH_* <#HSEHsp70_Hsf1_Hsf1_Hsf1_P> | 8.0E-2 s^-1^ |
| 25 | Hsp70 basal synthesis^c^ | Source→ Hsp70 | *k_basalsynHsp70_* | 8.0E-3 molecule s^-1^ |
| 26 | Hsp70 upregulation^c^ | HSEHsp70_Hsf1_Hsf1_Hsf1_P→ HSEHsp70_Hsf1_Hsf1_Hsf1_P  +Hsp70 | *k_upregHsp_* <#HSEHsp70_Hsf1_Hsf1_Hsf1_P> | 0.2 s^-1^ |
| 27 | Hsp70 proteasome binding^d^ | Hsp70+Proteasome→ Hsp70_Proteasome | *k_binHsp70Prot_*<#Hsp70><#Proteasome> | 1.2E-8 molecule^-1^ s^-1^ |
| 28 | Hsp70 degradation^c^ | ATP+Hsp70_Proteasome→ ADP+Proteasome | *k_degHsp70_*<#Hsp70_Proteasome> <#ATP>/(5000+<#ATP>) | 1.0E-2 s^-1^ |
| 29 | Hsp90 proteasome binding^d^ | Hsp90+Proteasome→ Hsp90_Proteasome | *k_binHsp90Prot_*<#Hsp90><#Proteasome> | 1.0E-8 molecule^-1^ s^-1^ |
| 30 | Hsp70 damage^c^ | Hsp70+ROS→ Hsp70_dam+ROS | *k_damHsp_*<#Hsp70><#ROS> | 1.0E-8 s^-1^ |
| 31 | MisP proteasome binding^c^ | Hsp70_MisP+Proteasome→ Hsp70+MisP_ Proteasome | *k_binMisPProt_* <#Hsp70_MisP><#Proteasome> | 1.0E-7 molecule^-1^ s^-1^ |
| 32 | MisP degradation | ATP+MisP_Proteasome→ ADP+Proteasome | *k_degMisp_* <#MisP_Proteasome>  <#ATP>/(5000+<#ATP>) | 1.0E-2 s^-1^ |
| 33 | Hsp70/PPX binding | Hsp70+Ppx→ Hsp70_Ppx | *k_binHsp70Ppx_<*#Hsp70><#Ppx> | 0.2 molecule^-1^ s^-1^ |
| 34 | Hsp70/PPX release | Hsp70_Ppx→ Hsp70+Ppx | *k_relHsp70Ppx_*<#Hsp70_Ppx> | 5.0 s^-1^ |
| 35 | Mkp1 synthesis | Source→Mkp1 | *k_synMkp1_* | 1.0E-5 molecule s^-1^ |
| 36 | Mkp1 proteasome binding | Mkp1+Proteasome→ Mkp1_Proteasome | *k_binMkp1Prot_<*#Mkp1>*<#*Proteasome*>* | 9.6E-9 molecule^-1^ s^-1^ |
| 37 | Mkp1 degradation | ATP+Mkp1_Proteasome→ ADP+Proteasome | *k_degMkp1_*<#Mkp1_Proteasome> <#ATP>/(5000+<#ATP>) | 1.0E-2 s^-1^ |
| 38 | Mkp1 activation | Mkp1+Hsp70→Mkp1_P+Hsp70 | *k_phosMkp1_*<#Mkp1><#Hsp70> | 2.0E-2 molecule^-1^ s^-1^ |
| 39 | Mkp1 inactivation | Mkp1_P+ROS→Mkp1+ROS | *k_dephosMkp1_<*#Mkp1_P><#ROS> | 1.0E-3 s^-1^ |
| 40 | JNK phosphorylation | Jnk+ROS→ Jnk_P+ROS | *k_phosJnk_*<#Jnk><#ROS> | 2.0E-2 molecule^-1^ s^-1^ |
| 41 | JNK de-phosphorylation | Jnk_P+Mkp1_P→ Jnk+Mkp1_P | *k_dephosJnkMkp1_*<#Jnk_P><#Mkp1_P> | 5.0E-2 molecule^-1^ s^-1^ |
| 42 | p38 phosphorylation | p38+ROS→ p38_P+ROS | *k_phosp38_*<#p38><#ROS> | 2.0E-2 molecule^-1^ s^-1^ |
| 43 | p38 de-phosphorylation | p38_P+Mkp1_P→ p38+Mkp1_P | *k_dephosp38Mkp1_*<#p38_P><#Mkp1_P> | 5.0E-2 molecule^-1^ s^-1^ |
| 44 | p38 ROS production | p38_P→p38_P+ROS | *k_genROSp38_*k_p38act_*<#p38_P> | 1.0E-4 s^-1^, 1.0 |
| 45 | Aggregation^e^ | 2 MisP→AggP | *k_agg_*<#MisP><#MisP-1>/2.0 | 1.0E-8 molecule^-1^ s^-1^ |
| 46 | Inclusion formation^e^ | MisP+AggP→SeqAggP | *k_agg_*<#MisP><#AggP> | 1.0E-8 molecule^-1^ s^-1^ |
| 47 | Inclusion growth^e^ | MisP+SeqAggP→2SeqAggP | *k_seqagg_*<#MisP><#SeqAggP> | 7.0E-7 molecule^-1^ s^-1^ |
| 48 | Proteasome inhibition by aggregates | AggP+Proteasome→ AggP_Proteasome | *k_binAggPProt_*<#AggP><#Proteasome> | 1.0E-5 molecule^-1^ s^-1^ |
| 49 | ROS generation | Source→ ROS | *k_genROS_* | 1.0E-2 molecule s^-1^ |
| 50 | ROS removal | ROS→ Sink | *k_remROS_* <#ROS> | 1.0E-3 s^-1^ |
| 51 | ROS generation by aggregates1 | AggP→ AggP+ROS | *k_genROSAggP_* <#AggP> | 1.0E-6 s^-1^ |
| 52 | ROS generation by aggregates2 | AggP_Proteasome→ AggP_Proteasome+ROS | *k_genROSAggP_* <#AggP_Proteasome> | 1.0E-6 s^-1^ |
| 53 | Akt Synthesis | Source→ Akt | *k_synAkt_* | 1.5E-3 molecule s^-1^ |
| 54 | Akt/Hsp90 binding | Akt+Hsp90→ Akt_Hsp90 | *k_binAktHsp90_* <#Akt><#Hsp90> | 3.7E-4 molecule^-1^  s^-1^ |
| 55 | Akt/Hsp90 release | Akt_Hsp90→ Akt+Hsp90 | *k_relAktHsp90_* <#Akt_Hsp90> | 7.0 s^-1^ |
| 56 | Akt/CHIP binding | Akt_Hsp90+CHIP→ Akt_CHIP_Hsp90 | *k_binCHIP_* <#Akt_Hsp90><#CHIP> | 2.0E-7molecule^-1^ s^-1^ |
| 57 | Akt/CHIP release | Akt_CHIP_Hsp90→ Akt_Hsp90+CHIP | *k_relCHIP_* <#Akt_CHIP_Hsp90> | 1.0E-8 s^-1^ |
| 58 | Akt Proteasome binding | Akt_CHIP_Hsp90+Proteasome→ Akt_Proteasome+CHIP+ Hsp90 | *k_binAktProt_* <#Akt_CHIP_Hsp90>  <#Proteasome> | 6.0E-8 molecule^-1^ s^-1^ |
| 59 | Akt/Proteasome release | Akt_Proteasome→ Akt+Proteasome | *k_relAktProt_* <#Akt_Proteasome> | 1.0E-8 s^-1^ |
| 60 | Akt Degradation | Akt_Proteasome+ATP→ Proteasome+ADP | *k_degAkt_* <#Akt_Proteasome>  <#ATP>/(5000+<#ATP>) | 1.0E-2 s^-1^ |
| 61 | p38 cell death | p38_P→p38_P+p38Death  +CellDeath^f^ | *k_p38death_* * *k_p38act_*<#p38_P> | 1.5E-7 s^-1^, 1.0 |
| 62 | JNK cell death | Jnk_P→Jnk_P+JNKDeath+ CellDeath^f^ | *k_Jnkdeath_*<#Jnk_P> | 1.5E-7 s^-1^ |
| 63 | PI cell death | AggP_Proteasome→ AggP_Proteasome+PIDeath+ CellDeath^f^ | *k_PIdeath_*<#AggP_Proteasome> | 2.0E-8 s^-1^ |

^a^ All reactions contain parameter *k_alive_* which is initially set to 1.0. ^b^Identical reaction takes place for HSEHsp90 with same value for the kinetic parameter. ^c^Identical reaction takes place for Hsp90 with same value for the kinetic parameter. ^d^Identical reactions occur for Hsp70_dam and Hsp90_dam. ^e^Identical reactions occur for Hsp70_dam and Hsp90_dam as for MisP with same value for the kinetic parameters. ^f^CellDeath is a dummy species which is required for the event DeathOfCell (Table 2).
