# Supplementary material for: Modelling the Role of the Hsp70/Hsp90 System in the Maintenance of Protein Homeostasis
Source: PLoS One. 2011 Jul 14;6(7):e22038. doi: 10.1371/journal.pone.0022038 (PMC3137010; doi:10.1371/journal.pone.0022038)

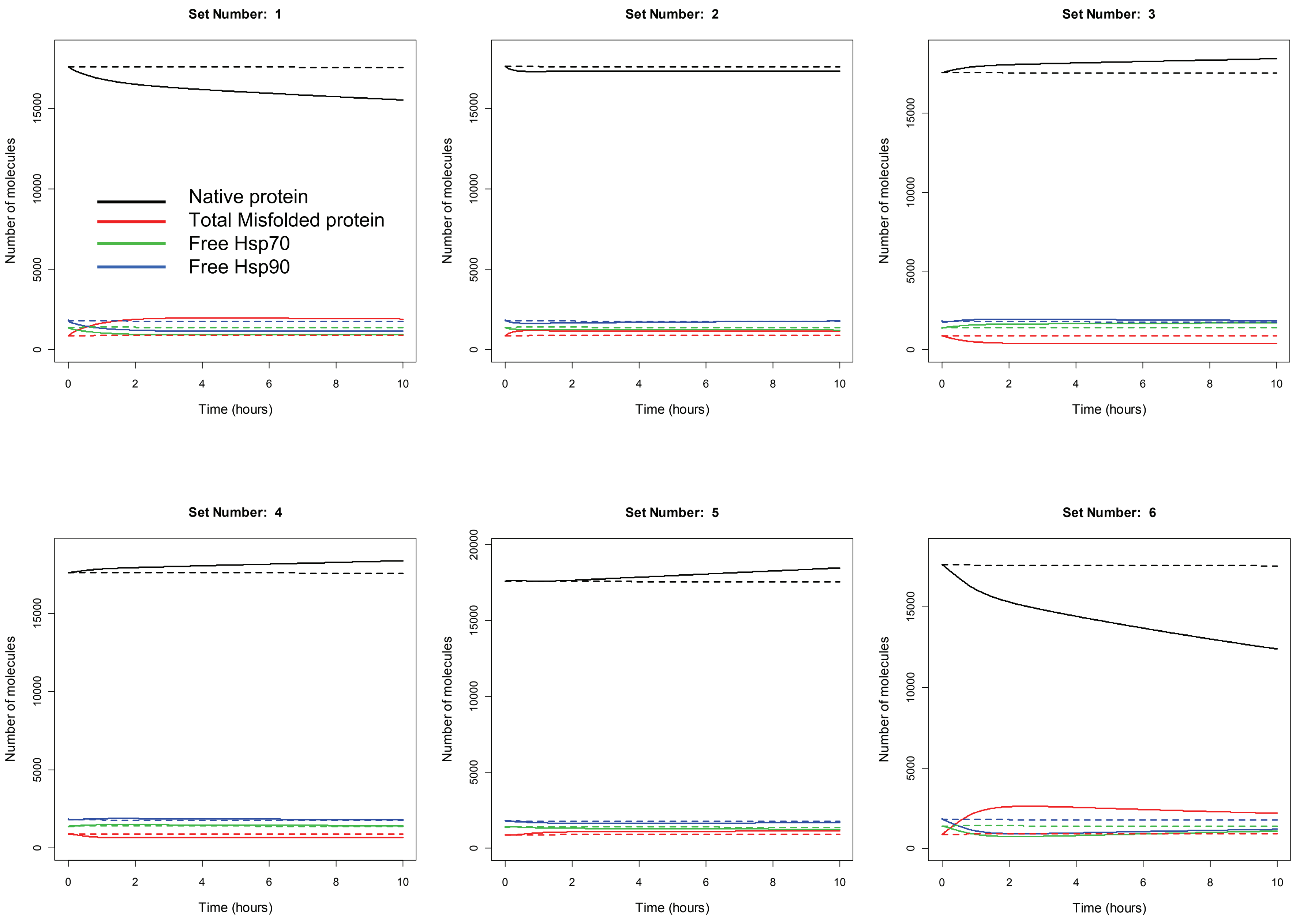
Set Number: 1
Set Number: 2
Set Number: 3
Set Number: 4
Set Number: 5
Set Number: 6
Native protein
Total Misfolded protein
Free Hsp70
Free Hsp90
Number of molecules
Time (hours)
0
5000
10000
15000
20000
2
4
6
8
10

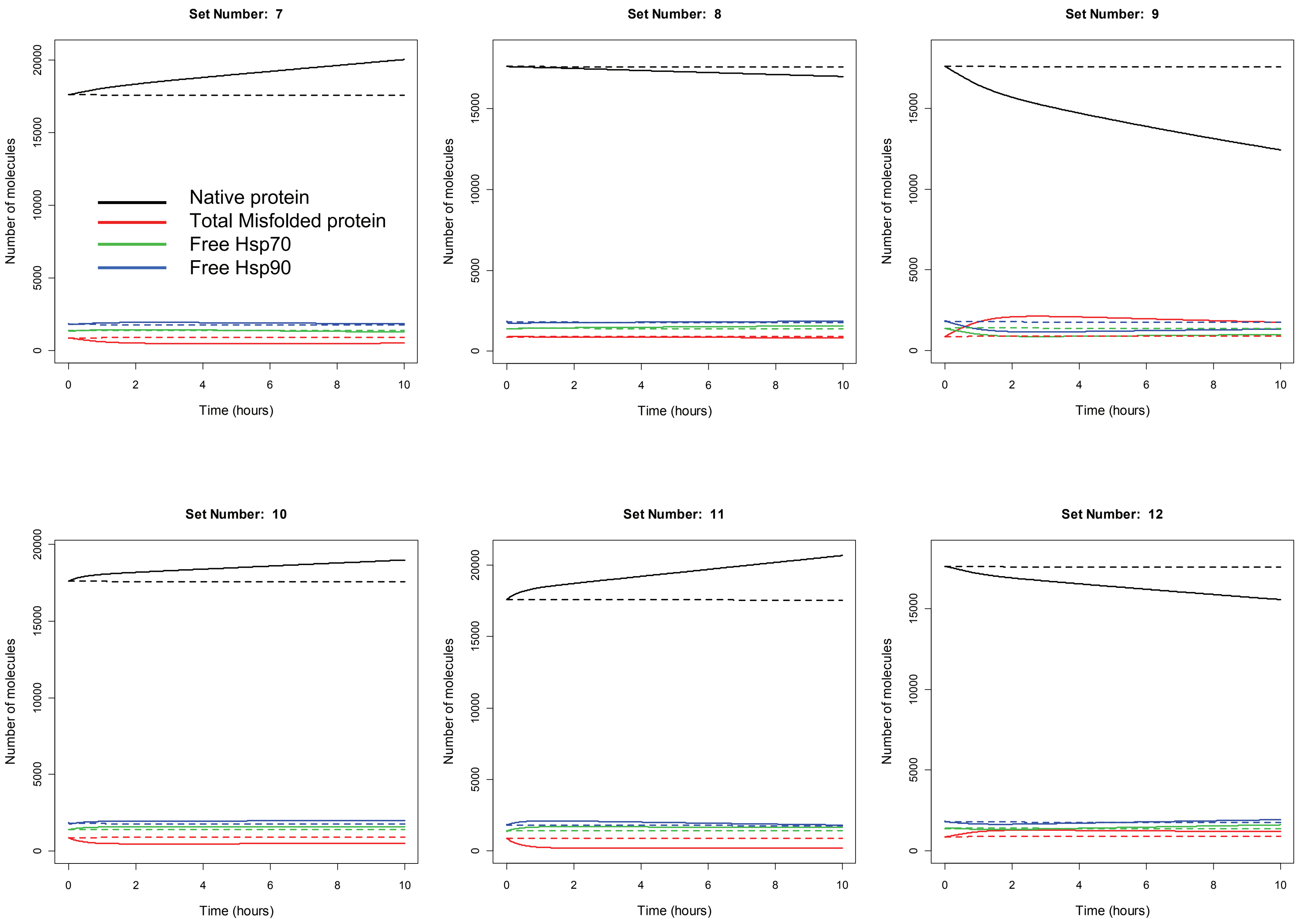
Set Number: 7
Set Number: 8
Set Number: 9
Set Number: 10
Set Number: 11
Set Number: 12
Native protein
Total Misfolded protein
Free Hsp70
Free Hsp90
Number of molecules
Time (hours)
0
5000
10000
15000
20000
0
2
4
6
8
10

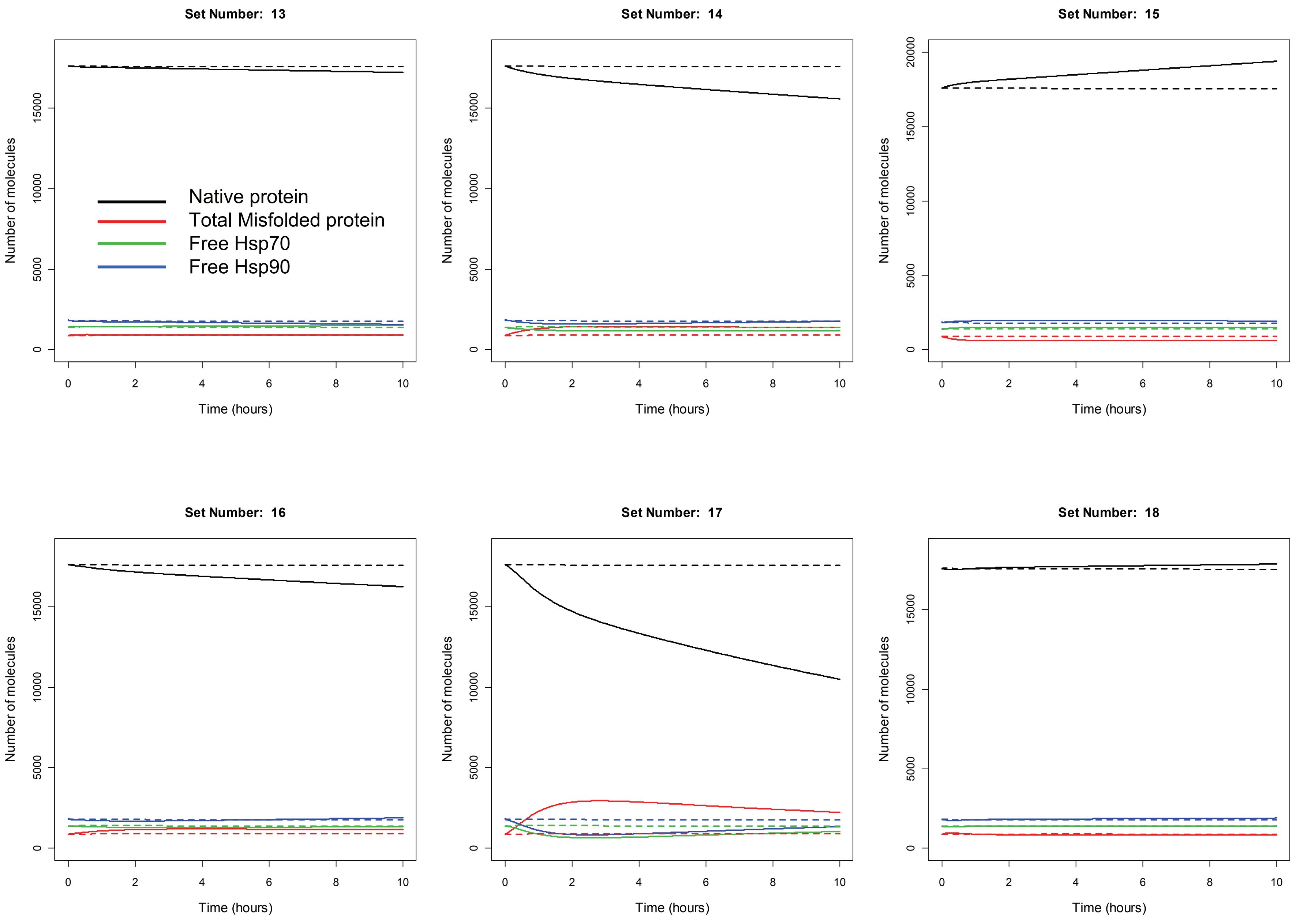
Set Number: 13
Native protein
Total Misfolded protein
Free Hsp70
Free Hsp90
Set Number: 14
Set Number: 15
Set Number: 16
Set Number: 17
Set Number: 18
Number of molecules
Time (hours)

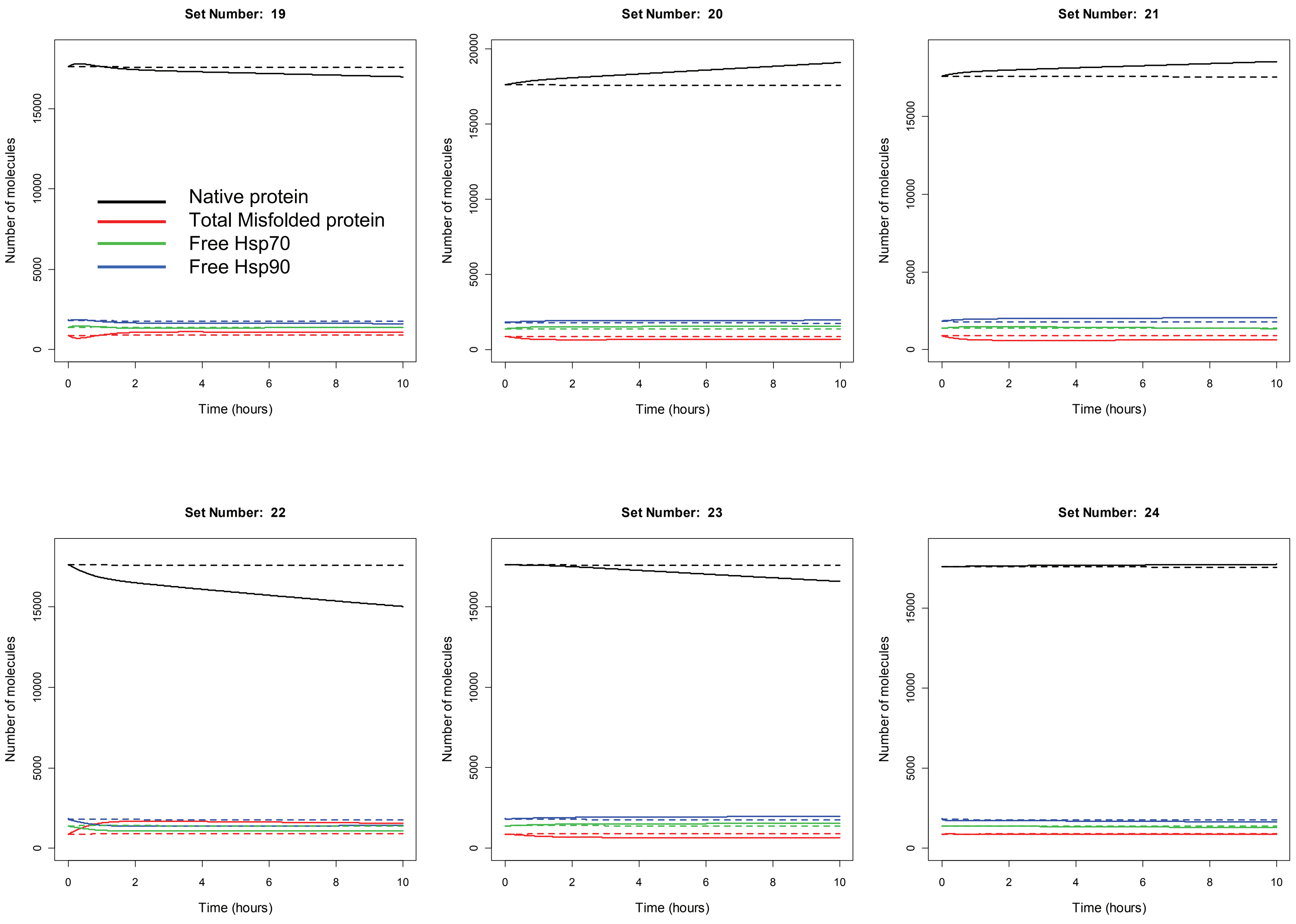

Set Number: 19
Set Number: 20
Set Number: 21
Set Number: 22
Set Number: 23
Set Number: 24
Native protein
Total Misfolded protein
Free Hsp70
Free Hsp90
Number of molecules
Time (hours)

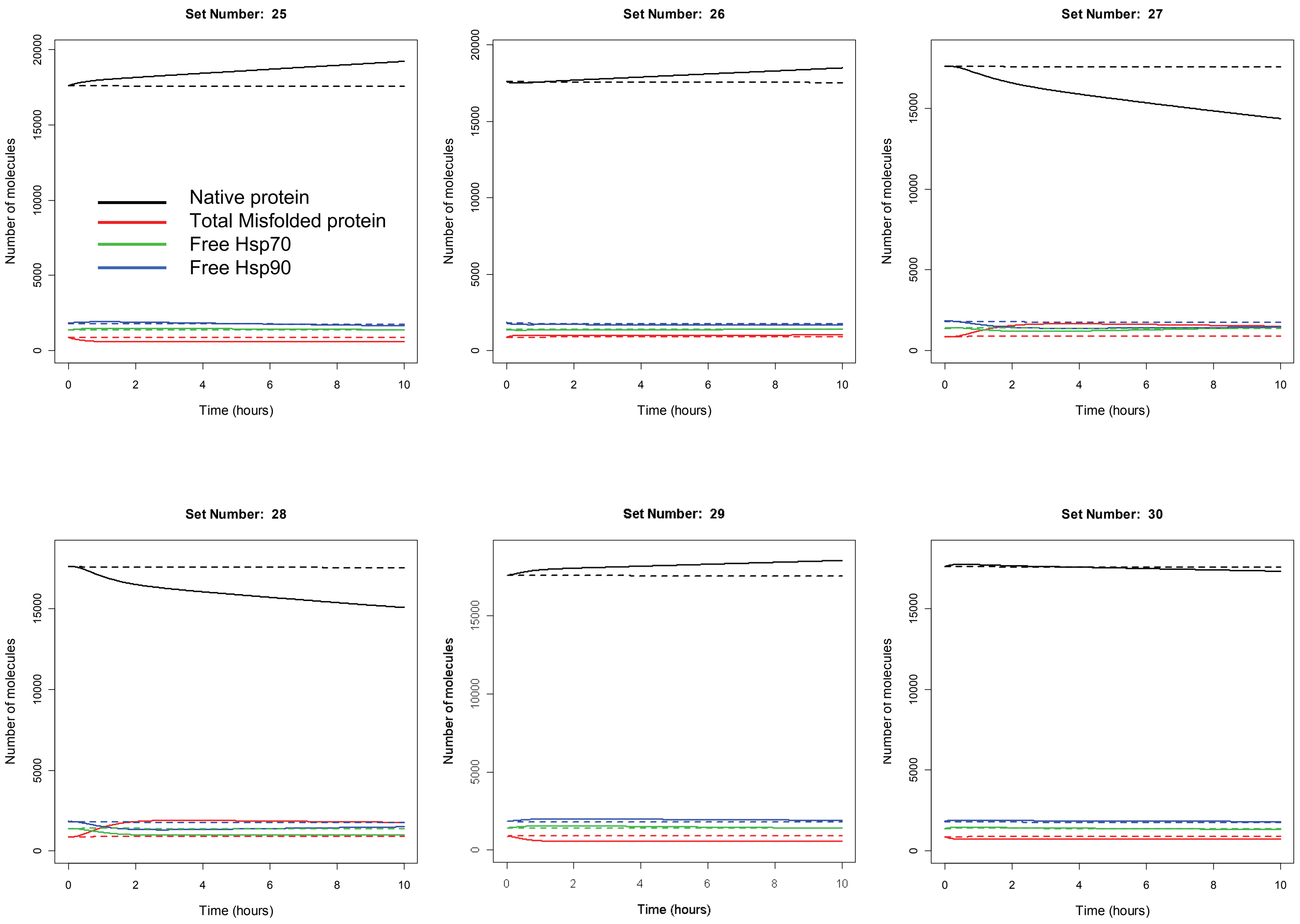
Set Number: 25
Set Number: 26
Set Number: 27
Set Number: 28
Set Number: 29
Set Number: 30
Native protein
Total Misfolded protein
Free Hsp70
Free Hsp90
Number of molecules
Time (hours)

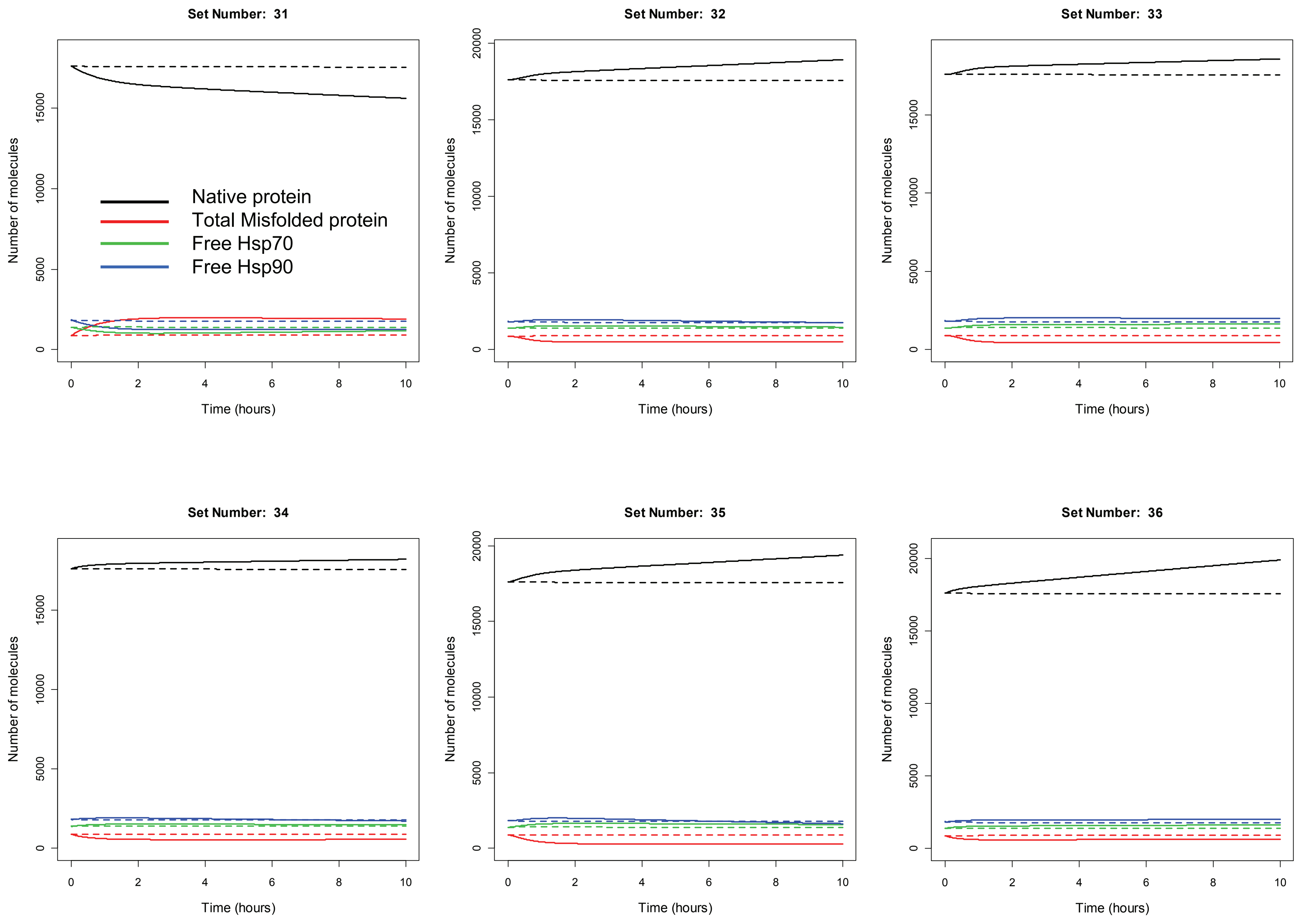
Set Number: 31
Set Number: 32
Set Number: 33
Set Number: 34
Set Number: 35
Set Number: 36
Native protein
Total Misfolded protein
Free Hsp70
Free Hsp90
Number of molecules
Time (hours)

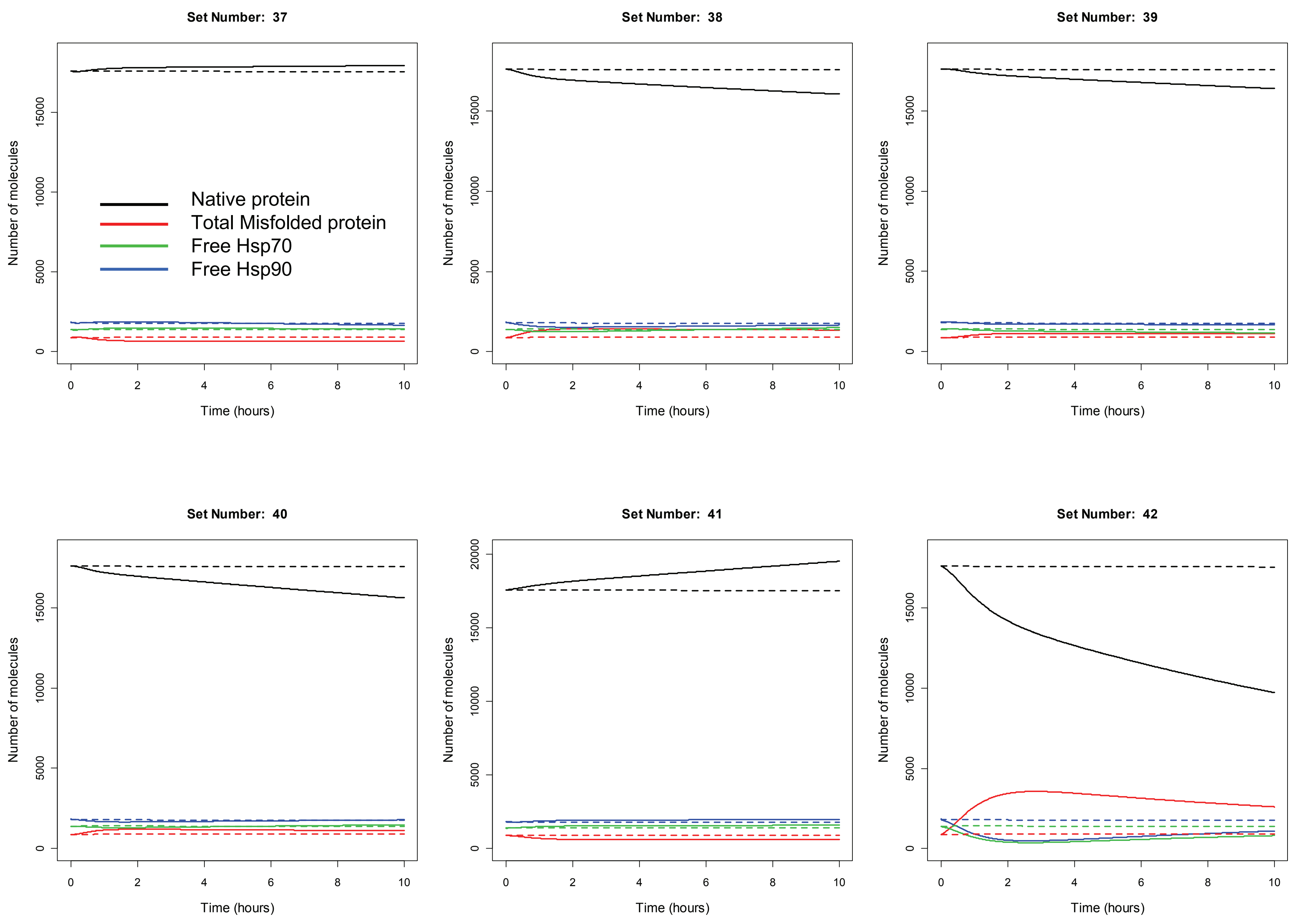
Set Number: 37
Set Number: 38
Set Number: 39
Set Number: 40
Set Number: 41
Set Number: 42
Native protein
Total Misfolded protein
Free Hsp70
Free Hsp90
Number of molecules
Time (hours)
0
5000
10000
15000
20000
0
2
4
6
8
10

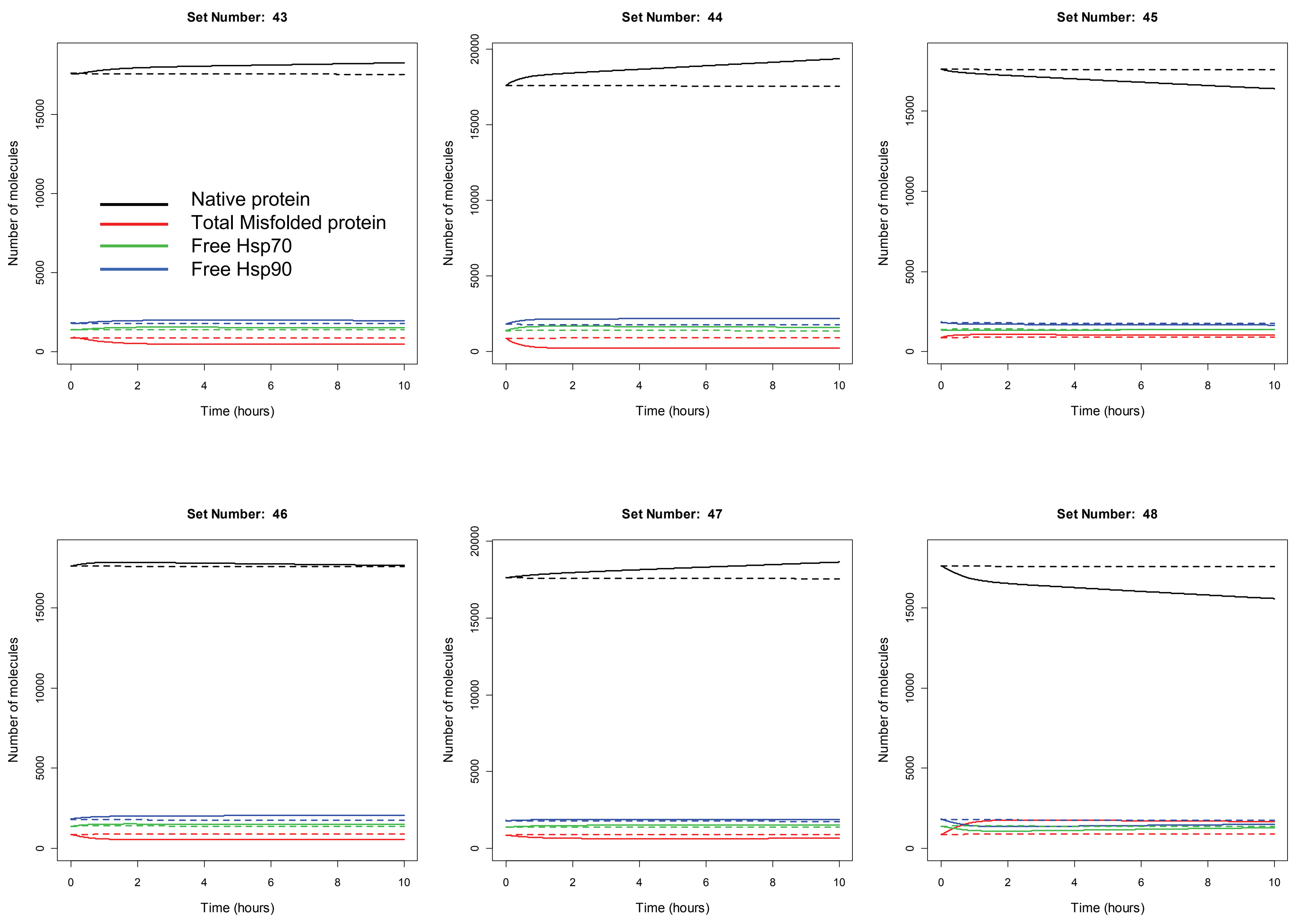

Set Number: 43
Set Number: 44
Set Number: 45
Set Number: 46
Set Number: 47
Set Number: 48
Native protein
Total Misfolded protein
Free Hsp70
Free Hsp90
Number of molecules
Time (hours)
0
5000
10000
15000
20000
0
2
4
6
8
10

Set Number: 49

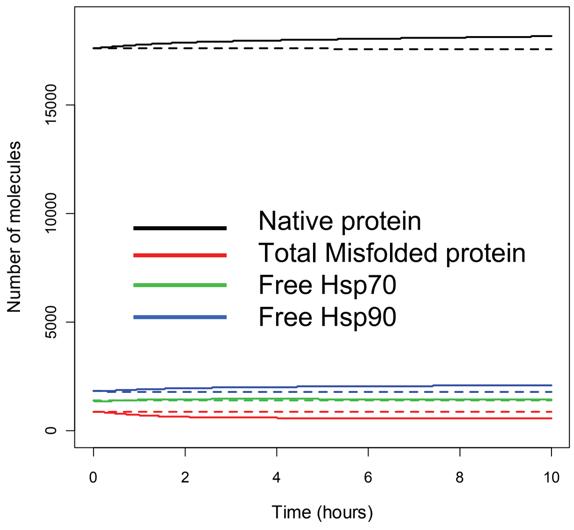

Set Number: 50

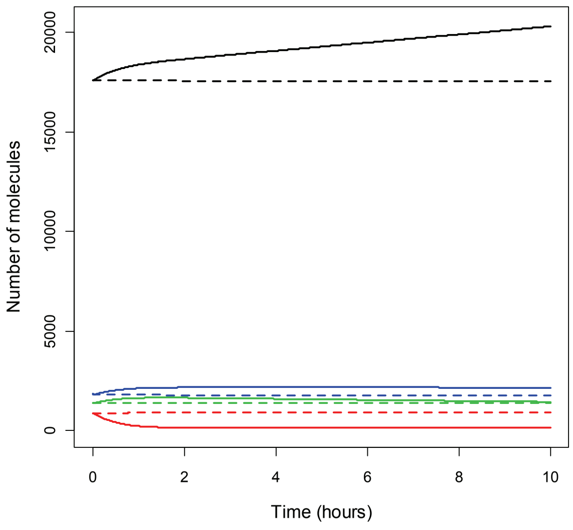

Supplement: Text S1 — Figures for the global parameter scan. Plots showing levels of native protein, misfolded protein and free pools of Hsp70 and Hsp90 for each of the 50 randomly chosen parameter sets. The number below each graph corresponds to the parameter set shown in Table S1. (PDF) [file pone.0022038.s011.pdf]
